# XRate: a fast prototyping, training and annotation tool for phylo-grammars

**DOI:** 10.1186/1471-2105-7-428

**Published:** 2006-10-03

**Authors:** Peter S Klosterman, Andrew V Uzilov, Yuri R Bendaña, Robert K Bradley, Sharon Chao, Carolin Kosiol, Nick Goldman, Ian Holmes

**Affiliations:** 1 Department of Bioengineering, University of California, Berkeley CA, USA; 2 European Bioinformatics Institute, Hinxton, Cambridgeshire, UK; 3 Department of Biological Statistics and Computational Biology, Cornell University, Ithaca NY, USA

## Abstract

**Background:**

Recent years have seen the emergence of genome annotation methods based on the *phylo-grammar*, a probabilistic model combining continuous-time Markov chains and stochastic grammars. Previously, phylo-grammars have required considerable effort to implement, limiting their adoption by computational biologists.

**Results:**

We have developed an open source software tool, xrate, for working with reversible, irreversible or parametric substitution models combined with stochastic context-free grammars. xrate efficiently estimates maximum-likelihood parameters and phylogenetic trees using a novel "phylo-EM" algorithm that we describe. The grammar is specified in an external configuration file, allowing users to design new grammars, estimate rate parameters from training data and annotate multiple sequence alignments without the need to recompile code from source. We have used xrate to measure codon substitution rates and predict protein and RNA secondary structures.

**Conclusion:**

Our results demonstrate that xrate estimates biologically meaningful rates and makes predictions whose accuracy is comparable to that of more specialized tools.

## Background

Hidden Markov models [HMMs], together with related probabilistic models such as stochastic context-free grammars [SCFGs], are the basis of many algorithms for the analysis of biological sequences [11,8,10,16]. An appealing feature of such models is that once the general structure of the model is specified, the parameters of the model can be estimated from representative "training data" with minimal user intervention (typically using the Expectation Maximization [EM] algorithm [14]). Combined with the continuous-time Markov chain theory of likelihood-based phylogeny, stochastic grammar approaches are finding similarly broad application in comparative sequence analysis, in particular the annotation of multiple alignments [83,26,53,46,74,80] (and, in some cases, simultaneous alignment and annotation [2,58]). This combined model has been dubbed the *phylo-grammar*. By contrast to the single-sequence case (for which there is much prior art in the field of computational linguistics [72,51]), the automated parameterization of phylo-grammars from training data is somewhat uncharted territory, partly because the application of the EM algorithm to phylogenetics is a recent addition to the theoretical toolbox. The phylo-grammar approaches that have been used to date have often used approximate and/or inefficient versions of EM to estimate parameters [59,81], or have been limited to particular subclasses of model, e.g. reversible or otherwise constrained models [9,38].

Previously, we showed how to apply the EM algorithm to estimate substitution rates in a phylogenetic reversible continuous-time Markov chain model [38]. This EM algorithm is exact and without approximation, using an eigenvector decomposition of the rate matrix to estimate summary statistics for the evolutionary history. We refer to this version of EM as "phylo-EM".

Here, we report several extensions to the phylo-EM method. Specifically, we give a version of the phylo-EM algorithm for the fully general, irreversible substitution model on a phylogenetic tree (noting that the irreversible model is a generalisation of the reversible case). We then present a flexible package for multiple alignment annotation using phylo-HMMs and phylo-SCFGs that implements these algorithms and is similar, in spirit, to the Dynamite package for generic dynamic programming using HMMs [5].

Using this package, it is extremely easy to design, train and apply a novel phylo-grammar, since new models can be loaded from an external, user-specified grammar file. Our hope is that the algorithms and software presented here will aid in the establishment of phylo-grammars in bioinformatics and that such methods will be as widely adopted for comparative genomics as HMMs and SCFGs have been.

## Overview

In 1981, Felsenstein published dynamic programming (DP) recursions for computing the likelihood of a phylogenetic tree for aligned sequence data, given an underlying substitution model [21]. Together with seminal papers by Neyman [64] and DayhofF *et al*.[12,13], this work heralded the widespread use probabilistic models in bioinformatics and molecular evolution. Felsenstein's underlying model is a finite-state continuous-time Markov chain, as described e.g. by Karlin and Taylor [43]. It is characterised by an instantaneous rate matrix **R **describing the instantaneous rates *R*_*ij *_of point substitutions from residue *i *to *j*. In the unifying language of contemporary "Machine Learning" approaches, Felsenstein's trees are recognisable as a form of graphical model [66] or factor graph [50], and the DP recursions an instance of the sum-product algorithm. (The connection to graphical models has been made more explicit with recent approaches that model other stochastic processes on phylogenetic trees, such as the evolution of molecular function [20].) Many parametric versions of this model have been explored, such as the "HKY85" model [32].

Beginning in the late 1980s, another class of probabilistic models for biological sequence analysis was developed. These models included HMMs for DNA [11] and proteins [8], and SCFGs for RNA [78,18]. Collectively, such models form a subset of the **stochastic grammars**. Originally used to annotate individual sequences, stochastic grammars were soon also combined with phylogenetic models to annotate alignments. Thus, trees have been combined with HMMs and/or SCFGs to predict genes [68] and conserved regions [23] in DNA sequences, secondary structures [83,26] and transmembrane topologies [53] in protein sequences, and basepairing structures in RNA sequences [46]. We refer to such hybrid models as **phylo-grammars**. Associated with these advances were novel methods to approximate context dependence of substitution models, such as CpG and other dinucleotide effects [81,55]. The phylo-grammars can also be viewed as a subclass of the "statistical alignment" grammars [34,37,60,36], which are derived from more rigorous assumptions about the underlying evolutionary model, including indels [84].

A compelling attraction of stochastic grammars (and probabilistic models in general) is that parameters can be systematically "learned" from data by maximum likelihood (ML). One reasonably good, general, albeit greedy and imperfect, approximation to ML is the EM algorithm [14]. EM applies to models which generate both "hidden" and "observed" data; e.g., the transcriptional/translational structure of a gene (hidden) and the raw genomic sequence (observed). The applications of EM to training HMMs (the Baum-Welch algorithm) [4] and SCFGs (Inside-Outside) [51] are well-established (reviewed in [16]), but what of phylo-grammars? While a limited version of EM for substitution models was published in 1996 [9,31], the full derivation for the general reversible rate matrix did not appear until 2002 [38]. The phylo-EM algorithm for rate matrices has since been further developed [94,35]. (Various alternatives to phylo-EM, such as eigenvector projections [3] and the "resolvent" [63], have also been used to estimate rate matrices; some approximate versions of phylo-EM have also been described [81,82].)

Conceptually, EM is straightforward: one simply alternates between imputing the hidden data (the "E-step") and optimizing the parameters (the "M-step"). The E-step typically results in a set of "expected counts" which are intuitively easy to interpret. (For example, the E-step for phylogenetic trees returns the number of times each substitution is expected to have occurred on each branch.) The EM algorithm has been intensely scrutinized and has been shown to be versatile, adaptable and fast [25,57], particularly the special case of phylo-EM [94]. We therefore argue that there are strong advantages to combining the form of EM used to train stochastic grammars (i.e. the Baum-Welch and Inside-Outside algorithms [16]) with the phylo-EM form used for parameterizing substitution models on phylogenetic trees [38].

## Previous applications of phylo-grammars

The program we have developed can handle a broad class of phylo-grammars within one framework. The following is a brief review of prior work that either uses phylo-grammars, or is ideally suited to the phylo-grammar framework.

This section is subclassified according to the complexity of the grammar, beginning with the simplest. Generally speaking, a phylo-grammar can be used to annotate a multiple sequence alignment in any context where a stochastic grammar could be used to annotate an individual sequence. The applications span DNA, RNA and protein sequence annotation.

### Point substitution models

A subset of the class of phylo-grammars is the class of homogeneous substitution models, where the mutation rate is not a function of position but rather is identical for every site. Such models can be represented as a single-state phylo-HMM. Examples include

**The Jukes-Cantor model **[41], **Kimura's two-parameter model **[44], **the HKY85 model **[32], **the general reversible model **[92], **and the general irreversible model **[91]. In the case of the Kimura and HKY85 models, the rate matrices are formulated para-metrically: that is, each substitution rate is expressed as a function of a small set of rate and/or probability parameters (e.g. in Kimura's model, there are two rate parameters: the transition rate and the transversion rate).

**Variable-rate models, where the evolutionary rate is allowed to vary from site to site **[90]. Yang used a finite number of discrete, fixed rate categories to approximate a continuous gamma distribution over site-specific rates. In essence, this can be viewed as special cases of the phylo-HMM of Felsenstein and Churchill [23], with the autocorrelation explicitly set to zero.

**Hidden-state models **[48,38]. A relative of the variable-rate model, the hidden-state model allows a variety of different substitution rate matrices to be used, depending on a hidden state variable that specifies the structural context of the site [48]. For example, a hydrophobically-inclined rate matrix might be used for buried amino acids and a hydrophilic matrix for exposed amino acids. An extension to the hidden-state model allows the hidden state variable itself to change over time at some slow rate, modeling rare changes in structural context [38]. An alternative extension allows correlations between hidden state variables at adjacent sites: this is essentially the idea behind the phylo-HMM, described below.

**Models for synonymous/nonsynonymous substitution ratio measurement; empirical rate matrices for codon evolution **[27,87]. Codon substitution matrices such as WAG [87] can be used to measure the ratio *r *of synonymous to nonsynonymous substitution rates, which may be indicative of purifying (*r *< 1), neutral (*r *= 1) or diversifying (*r *> 1) selection. These models are also related to the exon prediction phylo-HMMs in EVOGENE [68] and EXONIPHY [80], described below.

**Amino acid substitution models **[12,28]. Likelihood calculations using these models can, as with the other substitution models discussed above, be viewed as trivial applications of phylo-grammars.

**Context-sensitive substitution models **[81]. Siepel and Haussler introduced several alternate approximations for calculating the likelihood of alignments assuming a nearest neighbor substitution model, suitable for capturing the context-sensitivity of the substitution process that is observed in real sequence alignments (most notoriously in genomes wherein CpG methylation is used as a mechanism of epigenetic regulation, leading to elevated rates for the mutations CpG→TpG and CpG→ApG). Siepel and Haussler's method ignores longer-range correlations induced by nearest-neighbor effects, but is effective in practice. (It may be regarded as an approximation to the more rigorous analysis of Lunter and Hein [55].)

**Figure 1:**
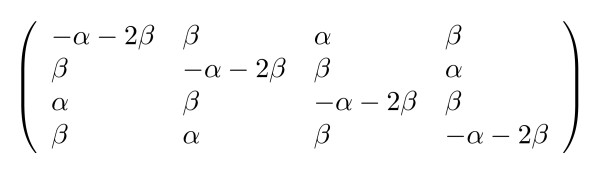
Kimura's two-parameter model. The state order is {*A*, *C*, *G*, *T*}. Each entry is a function of the reduced parameter set (*α*, *β*) where *α *and *β *are rates.

**Figure 2:**
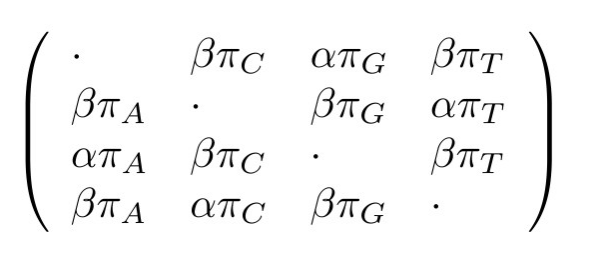
Hasegawa *et al*'s six-parameter model. The state order is {*A*, *C*, *G*, *T*}. The negative on-diagonal elements have been omitted for brevity (they are constrained by the requirement that each row sums to zero). Each entry is a function of the reduced parameter set (*α*, *β*, *π*_*A*_, *π*_*C*_, *π*_*G*_, *π*_*T*_) where (*α*, *β*) are rates and (*π*_*A*_, *π*_*C*_, *π*_*G*_, *π*_*T*_) are probabilities.

Many of these models can be expressed using the **General Parametric Substitution Model**, which we define as the substitution model wherein all substitution rates and initial probabilities can be expressed as simple functions of a (reduced) set of rate and probability parameters. As an example, Kimura's two-parameter model [44] is shown (see figure 1) along with the HKY85 six-parameter model [32] (see figure 2).

As long as each parameter in a parametric substitution model can be interpreted either as a rate (such as Kimura's transition and transversion rates) or a probability (such as the HKY85 equilibrium distribution over nucleotides), the phylo-EM algorithm can be adapted to estimate such parameters via the computation of expected event counts. A formal description of the sets of allowable rate and probability functions is given in the Supplementary Material [see Additional file 1].

Although the particular models used above (Kimura and HKY85) are reversible, matrices of allowable rate functions can in general be irreversible. Our General Parametric Model may thus be regarded as a generalisation of the General Irreversible Model.

### Phylo-HMMs

Phylo-HMMs form a class of models slightly more complex than point substitution models. In a phylo-HMM, each column (or group of adjacent columns) is associated with a hidden state, representing the evolutionary context of the site. Each hidden state is conditionally dependent upon the immediately preceding state (the Markov property).

Tasks that have been addressed using phylo-HMMs include:

**Measurement of variation of evolutionary rate among sites in DNA **[23]. Felsenstein and Churchill construct an HMM with three states. Each state generates an alignment column according to a point substitution process on a tree [21]. The overall evolutionary rate for the column depends on the state from which it is emitted: each state thus corresponds to a "rate category" (the relative rates for the three states are 0.3, 2.0 and 10.0). The use of an HMM allows for an autocorrelated model of rate variation.

**Modeling site-specific residue usage in proteins**[9,31]. While site-specific profiles are familiar tools in bioinformatics, early tools such as Gribskov profiles [29] and hidden Markov models [8] ignored phylogenetic correlations in the dataset, leading to biased sampling. Phylo-grammars incorporate these correlations directly. In these papers, Bruno *et al*. introduced an initial EM algorithm for estimating rate matrices.

**Prediction of secondary structure in proteins**[83,26]. In a similar manner to Felsenstein and Churchill, a three-state HMM is constructed wherein each state emits an alignment column using a substitution rate matrix. Here, however, the states correspond to different units of secondary structure (loop, *α*-helix and *β*-sheet). The substitution rate matrix for each state reflects the frequency distribution and substitution patterns for that secondary structural class. The method performs less well than established secondary structure prediction algorithms, but shows promise, in particular given the simplicity of the model (three states only). Later work expanded the number of states in the phylo-HMM to eight (correspondingly increasing the number of parameters). Note that, as more parameters are introduced into this kind of phylo-HMM, the problem of "training" those parameters grows in importance.

**Prediction of exons and protein-coding gene structures in DNA **[68,80]. The basis for the gene prediction programs EVOGENE and EXONIPHY, respectively, these phylo-HMMs are based on substitution models for codon triplets with 4^3 ^= 64 states. The paper by Siepel and Haussler introduced the term "phylo-HMM" and used an approximate version of the EM algorithm introduced by Holmes and Rubin for parameterization [38].

**Detection, modeling and annotation of transcription factor binding sites in DNA **[62]. Here, the EM algorithm and other formulae of Bruno and Halpern [9,31] is used to model site-specific residue frequencies in alignments of promoter regions (rather than proteins, as addressed by Bruno and Halpern).

**Detection of conserved regions in multiple alignments of genomic DNA **[79]. Phylo-HMMs to detect conserved regions can be viewed as extensions of Felsenstein and Churchill's original model with more rate categories. This approach has been used to detect highly-conserved regions in vertebrate, insect, nematode and yeast genomes. Approaches measuring the substitution rate per site [79,85], the local indel rate [54] and/or the CpG mutation bias [81,55] have all shown merit.

Analogously to some of the point substitution models, many phylo-HMMs can be expressed parametrically. An example of such a model is the one used by Siepel's PHASTCONS program, whose phylo-HMM has ten states ranging from slow to fast overall substitution rate. Moving from one state to another, the *relative *substitution rates between different nucleotides do not change (i.e. the ratio *R*_*ij*_/*R*_*kl *_is constant for any *i*, *j*, *k*, *l *∈ {*A*, *C*, *G*, *T*}); only the *overall *substitution rate varies (i.e. the absolute value *R*_*ij *_is not constant). Such consistency across states can be achieved by writing the rate matrices for the ten states as *k*_1_**R**, *k*_2 _× **R**, *k*_3 _× **R**... *k*_10 _× **R **where the *k*_*i *_are scalar multipliers and **R **is a relative rate matrix shared by all the states. Similarly, the rate matrices of Felsenstein and Churchill's three-state phylo-HMM can be written 0.3 × **R**, 2 × **R **and 10 × **R**. Both are examples of the general parametric phylo-HMM.

### Phylo-SCFGs

The most complex class of phylo-grammar considered here is the phylo-SCFG. Most commonly used to model RNA secondary structure, these grammars are capable of modeling covariation between paired sites. In an SCFG, covarying sites must be strictly nested, allowing the modeling of foldback structures but not pseudoknots, kissing loops or other topologi-cally elaborate RNA structures [45].

Tasks that have been addressed using phylo-SCFGs include:

**Prediction of RNA secondary structure **[46,47]. The Pfold program in this paper introduced the first phylo-SCFG, combining stochastic context-free grammars (used to model RNA structure) with evolutionary substitution models. Since HMMs are a subset of SCFGs, the framework of phylo-SCFGs includes the previously discussed phylo-HMMs. The Pfold program also allowed for user-specified grammars; however, it lacked a fast EM-like algorithm for estimating grammar parameters from data (by contrast, the non-phylogenetic SCFGs used elsewhere in bioinformatics can be rapidly trained using the Inside-Outside algorithm [16]). A key feature of these models is the use of 16-state "basepair models" for modeling the simultaneous coevolution of functional base-pairs in RNA structures. Again, fast and effective parameterization of the model is an important issue.

**Detection of noncoding RNA genes **[67]. A similar model to Pfold was used by the Evofold program, which uses a phylo-SCFG to parse genomic alignments into noncoding RNA and other features [67].

**Detection of RNA secondary structure within exons **[69]. The RNA-Decoder program uses a parametric phylo-SCFG to model exonic regions in which there is simultaneous selection on both the translated protein sequence and the secondary structure of the pre-mRNA. Such regions have been found in viral genomes and hypothesized to fulfil a regulatory role [69]. Due to the complexity of these models and the sparsity of training data, parametric rate functions are required to limit the number of free parameters that must be estimated.

**Detection of accelerated selection in human noncoding RNA **[70]. Pollard *et al *used phylo-HMMs and phylo-SCFGs to identify a neurally-expressed RNA gene, HARF1, that had undergone recent accelerated evolution in the lineage separating humans from the human-chimp ancestor.

## Implementation

In practice, users of phylo-grammars need to do a similar core set of tasks in order to perform data analysis. These tasks may include model development, structured parameterization, estimation of parameter values and application of the model to annotate alignments. Using the framework of phylo-grammars, an implementation enabling all these tasks is possible. The EM algorithm provides a general and consistent approach to parameter estimation, while standard "parsing" algorithms (the Viterbi and Cocke-Younger-Kasami (CYK) algorithms [16]) address the problem of annotation.

We have implemented EM and Viterbi/CYK parsing algorithms in our software. The general irreversible phylo-EM algorithm, using eigenvector decompositions, is described in the Supplementary Material to this paper [see Additional file 1]. (Note that this model is more general than the "general reversible model" [92], which can be regarded as a special case wherein the rates obey a detailed balance symmetry so that *π*_*i*_*R*_*ij *_= *π*_*j*_*R*_*ji*_.) The main advance over previous descriptions of this algorithm [38,81] is a complete closed-form solution for the M-step of EM for irreversible models, including a full algebraic treatment of the complex conjugate eigenvector pairs [see Additional file 1]. This closed-form solution for the M-step eliminates the need for numerical optimization code as part of EM. The Viterbi and CYK algorithms are described in full elsewhere [16].

The essential idea of EM is iteratively to maximize the *expected log-likelihood *with respect to the rate parameters, where the expectation is taken over the posterior distribution of the missing data using the current parameters. In the case of phylo-EM, the missing data are the sequences ancestral to the observed sequence data.

As with many instances of EM, the posterior distribution over the missing data in phylo-EM can be summarized via a representative set of "counts" that, being expectations, have convenient additive properties.

These counts have the following intuitive meaning with respect to the ancestral states of the evolutionary process: (i) the expected residue composition at the root node of the tree; (ii) the expected number of times each type of point mutation occurred; (iii) the expected amount of evolutionary time each residue was extant.

Each of these counts is summed over all branches of the phylogenetic tree and then over all columns in the alignment (or groups of columns). The sum over columns is weighted by the posterior probability that each column (or group of columns) was generated by a particular state.

Note that it is relatively easy to obtain naive estimates for the phylo-EM counts (e.g. using parsimony), but that such naive estimates are in general systematically biased. In particular, they tend to underestimate the number of substitutions that actually occurred.

A stochastic grammar consists of a set of "nonterminal" symbols (equivalent to the "states" of an HMM), a set of "terminal" symbols and a set of "production rules" for transforming nonterminals. In a context-free grammar, each production rule transforms a single nonterminal into a (possibly empty) sequence of terminals and/or nonterminals. The iterative application of such rules can be represented as a tree structure known as the "parse tree" [16]. In biological applications, there is typically a large number of parse trees that can explain the observed data. This contrasts with applications in computational linguistics, where there are typically only a small number of parses consistent with the data.

To apply EM to a stochastic grammar, one must compute the expected number of times each production rule was used in the derivation of the observed alignment. These expected counts are summed over the posterior distribution of parse trees, and are calculated using the Inside-Outside algorithm.

The set of terminal symbols for a phylo-grammar is the set of possible alignment columns (in contrast to a single-sequence grammar, where the set of terminal symbols corresponds to the residue alphabet). The phylo-EM algorithm is used to estimate the rate parameters associated with the emission of these symbols by the grammar.

### Programs

The following open source software tools, implementing the algorithms and models described in this paper, are freely available (see Availability and Requirements).

xgram – a implementation of the EM algorithm for training phylo-grammars, i.e. the Inside-Outside and Forward-Backward algorithms combined with the EM algorithm for the general irreversible (and reversible) substitution models. This program implements the general irreversible EM algorithm described in the Supplementary Material [see Additional file 1], along with the general reversible EM algorithm described previously [38]. The grammar can be user-specified via an extensible file format, described below. Parametric grammars are allowed (so that individual substitution rates and/or rule probabilities can be constrained to arbitrary functions of a smaller set of model parameters). The xgram tool is capable of reproducing most of the phylo-grammar models listed in this paper. In its generic applicability, xgram is similar to the dynamic programming engine Dynamite [5], although the class of models is different (phylo-grammars *vs *single- and pair-HMMs) and the functionality broader (including parameterization by phylo-EM, as well as Viterbi and CYK annotation codes). Also included is an implementation of the neighbor-joining algorithm for fast estimation of tree topologies [77], and another version of the EM algorithm for rapidly optimising branch lengths of trees with fixed topology [24]. The model underlying xgram also allows for dynamically evolving "hidden states" associated with each site, again as previously described [38].

xrate – a version of xgram including several "preset" grammars for point substitution models, including the general irreversible and reversible substitution models.

xfold – a version of xgram including several "preset" grammars for RNA analysis, including that of the Pfold program [46].

xprot – a version of xgram including several "preset" grammars for protein analysis, including a grammar similar to that used by Thorne *et al*. for protein secondary structure prediction [83].

All of the above programs can be driven by any user-specified phylo-grammar. Having specified a grammar, or chosen one of the presets, the user can

• Estimate the ML parameterization of the grammar for the training set via EM, using Inside-Outside or Forward-Backward algorithms (auto-selected by program) [16], together with the phylo-EM algorithm described in the Supplementary Material [see Additional file 1];

• Find the maximum likelihood (ML) parse tree, using Cocke-Younger-Kasami (CYK) or Viterbi algorithms (auto-selected by program) [16], with phylogenetic likelihoods calculated by pruning [21];

• Annotate the alignment, column-by-column, with user-specified labels, using the ML parse tree;

• Find the posterior probability of each node in the ML parse tree.

The parse tree can also be constrained, completely or partially, by including complete or partial annotations in the input alignment. For example, one can annotate several known examples of a TF binding site in a multiple alignment. One can then allow the grammar to "learn" these examples and predict new binding sites.

### File formats

The input and output format for sequence alignment data is the Stockholm format, as used by PFAM and RFAM. The wildcard character is the period ".". Annotation of columns with the wildcard character allows for incompletely labeled data and hence partially supervised learning. If a given annotation is specified in the grammar but absent from the training data, it will be treated as a string of wildcards and all compatible possibilities will be summed over.

Any phylo-grammar can be specified, using a format based on LISP S-expressions [56,75]. The format is human-readable and succinct, while being machine-parseable and extensible.

Phylo-grammar specification files contain several elements:

• An *alphabet*, describing valid sequence tokens (e.g. nucleotides or amino acids) along with any degenerate or (in the case of nucleotides) complementary tokens.

• One or more *chains*, each describing a finite-state continuous-time Markov chain, including rate parameters;

• Optionally (for parametric models) a set of rate and probability *parameter values*;

• A set of *transformation rules*, which also serve to define the nonterminals in the grammar.

**Figure 3:**
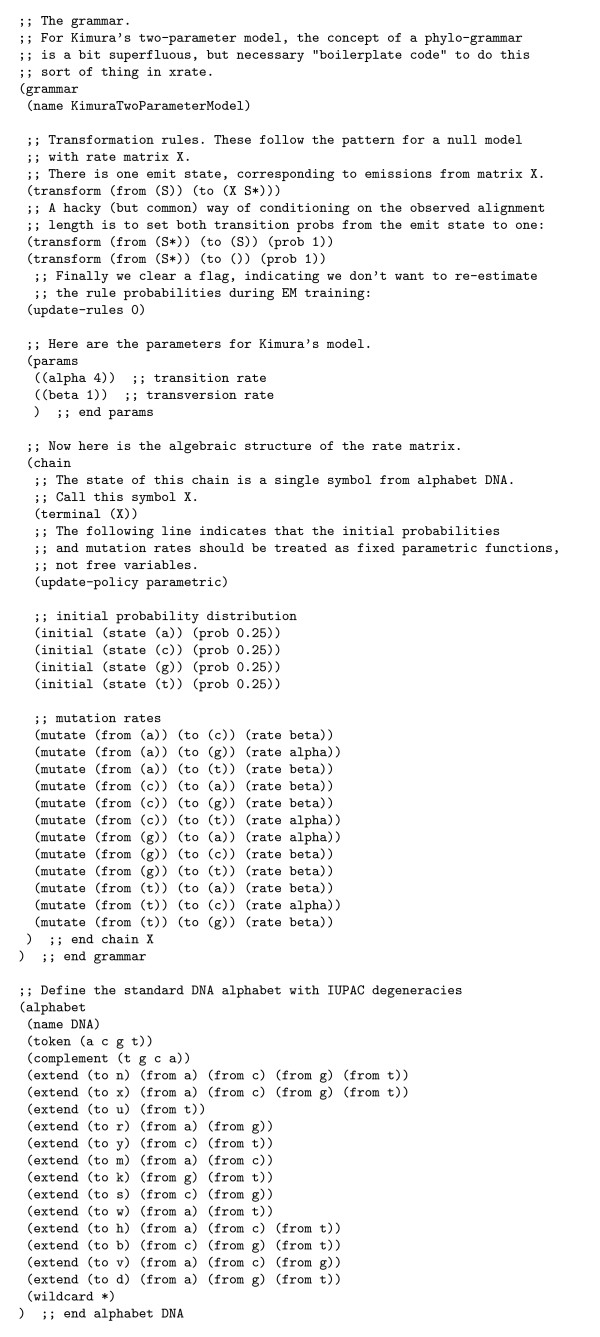
An xgram-format grammar for Kimura's two-parameter model.

As an example, the grammar for the Kimura two-parameter rate matrix is shown (see figure 3). A more complete and up-to-date description of the format can be found online [88], as can discussion of the latest version of xrate and its companion programs [89].

## Results and discussion

We illustrate the potential of xrate as a quick tool for prototyping phylo-grammars by re-implementing several prior applications and testing on real and simulated data. As applications we choose firstly a codon substitution model which is both computationally intensive and parameter-rich (due to the size of the rate matrix). Secondly, we compare xrate's performance in predicting protein structure to a previously used phylo-HMM. Thirdly, we compare xrate to a previously used phylo-SCFG for predicting RNA secondary structure.

To visualize rate matrices, we use figures that we refer to as "bubble-plots" (see figure 11). In a bubbleplot, the area of a circle in the main matrix is proportional to the rate of the corresponding substitution, with the grey circle in the upper-left repesenting the scale. The offset row shows the equilibrium probability distribution over states: here, the area of a circle is proportional to the equilibrium probability of the corresponding state. Additional color-coding is used on a case-by-case basis.

### Fitting codon models

In the past, various amino acid substitution models have been estimated using ML techniques (e.g., mtREV [15], WAG [87]). An ML estimation of codon substitution models, however, has seemed infeasible for a long time because of the computational burden involved with such parameter-rich models. This section shows that xrate is capable of tackling the problem. The full results of a particular study are being published elsewhere (Kosiol, Holmes and Goldman, in prep.); here, we will restrict attention to simulation results showing that xrate can do these sorts of analyses reliably.

The number of independent parameters for a reversible substitution model with *N *character states can be calculated as N(N+1)2−2
 MathType@MTEF@5@5@+=feaafiart1ev1aaatCvAUfKttLearuWrP9MDH5MBPbIqV92AaeXatLxBI9gBaebbnrfifHhDYfgasaacH8akY=wiFfYdH8Gipec8Eeeu0xXdbba9frFj0=OqFfea0dXdd9vqai=hGuQ8kuc9pgc9s8qqaq=dirpe0xb9q8qiLsFr0=vr0=vr0dc8meaabaqaciaacaGaaeqabaqabeGadaaakeaadaWcaaqaaiabd6eaojabcIcaOiabd6eaojabgUcaRiabigdaXiabcMcaPaqaaiabikdaYaaacqGHsislcqaIYaGmaaa@355B@. This means that for the estimation of a 20-state amino acid model, 208 independent parameters need to be calculated. In contrast, to estimate a 61-state codon model (excluding stop codons), 1889 independent parameters have to be determined.

To test the robustness of xrate's ability to fit parameter-rich models to aligned sequence data, we simulated a data set using all phylogenies of the Pandit database of protein domain alignments [86], using a standard model of codon evolution (the MO model [93] [see Additional file 1]). In this model, rates of substitutions involving changes to multiple nucleotides are zero, so that the rate matrix is sparsely populated.

xrate is able to recover M0 well from this 'artifical' Pandit database. The true rates used in the simulation are shown (see figure 8). These may be compared with the recovered rates (see figure 9).

A scatter plot of true *vs *estimated rates allows a more detailed analysis (see figure 10). This plot shows the true instantaneous rates qij(true)
 MathType@MTEF@5@5@+=feaafiart1ev1aaatCvAUfKttLearuWrP9MDH5MBPbIqV92AaeXatLxBI9gBaebbnrfifHhDYfgasaacH8akY=wiFfYdH8Gipec8Eeeu0xXdbba9frFj0=OqFfea0dXdd9vqai=hGuQ8kuc9pgc9s8qqaq=dirpe0xb9q8qiLsFr0=vr0=vr0dc8meaabaqaciaacaGaaeqabaqabeGadaaakeaacqWGXbqCdaqhaaWcbaGaemyAaKMaemOAaOgabaGaeiikaGIaemiDaqNaemOCaiNaemyDauNaemyzauMaeiykaKcaaaaa@3852@ of M0 plotted versus the instantaneous rates qij(est)
 MathType@MTEF@5@5@+=feaafiart1ev1aaatCvAUfKttLearuWrP9MDH5MBPbIqV92AaeXatLxBI9gBaebbnrfifHhDYfgasaacH8akY=wiFfYdH8Gipec8Eeeu0xXdbba9frFj0=OqFfea0dXdd9vqai=hGuQ8kuc9pgc9s8qqaq=dirpe0xb9q8qiLsFr0=vr0=vr0dc8meaabaqaciaacaGaaeqabaqabeGadaaakeaacqWGXbqCdaqhaaWcbaGaemyAaKMaemOAaOgabaGaeiikaGIaemyzauMaem4CamNaemiDaqNaeiykaKcaaaaa@36E1@ estimated from data simulated from M0. If qij(true)
 MathType@MTEF@5@5@+=feaafiart1ev1aaatCvAUfKttLearuWrP9MDH5MBPbIqV92AaeXatLxBI9gBaebbnrfifHhDYfgasaacH8akY=wiFfYdH8Gipec8Eeeu0xXdbba9frFj0=OqFfea0dXdd9vqai=hGuQ8kuc9pgc9s8qqaq=dirpe0xb9q8qiLsFr0=vr0=vr0dc8meaabaqaciaacaGaaeqabaqabeGadaaakeaacqWGXbqCdaqhaaWcbaGaemyAaKMaemOAaOgabaGaeiikaGIaemiDaqNaemOCaiNaemyDauNaemyzauMaeiykaKcaaaaa@3852@ = qij(est)
 MathType@MTEF@5@5@+=feaafiart1ev1aaatCvAUfKttLearuWrP9MDH5MBPbIqV92AaeXatLxBI9gBaebbnrfifHhDYfgasaacH8akY=wiFfYdH8Gipec8Eeeu0xXdbba9frFj0=OqFfea0dXdd9vqai=hGuQ8kuc9pgc9s8qqaq=dirpe0xb9q8qiLsFr0=vr0=vr0dc8meaabaqaciaacaGaaeqabaqabeGadaaakeaacqWGXbqCdaqhaaWcbaGaemyAaKMaemOAaOgabaGaeiikaGIaemyzauMaem4CamNaemiDaqNaeiykaKcaaaaa@36E1@ the points would lie on the bisection line *y *= *x*. Thus the deviation of the points from the bisection line indicates how different the rates are.

If one is interested in drawing biological conclusions from the estimated rate parameters, then it is of interest to consider xrate's estimates of rates which are zero in the true model, xrate sometimes inferred erroneously very small non-zero values for the instantaneous rates of double and triple changes from the simulated data set (in the M0 model, which was used to generate the data, such substitutions have zero rate). However, this error can be correctly identified by comparing log-likelihoods calculated by xrate under the following nested models: For the general model allowing for single, double and triple nucleotide changes 1889 parameters had to be estimated. The best likelihood calculated for general estimation is In *L*_*general *_= -28930383.06. Using xrate we can also restrict the rate matrices to single nucleotide changes only. For this model 322 parameters had to be estimated. The best likelihood calculated for restricted estimation is lnL_*restricted *_= -28930894.86.

Although the log-likelihood for the general rate matrix allowing for single, double and triple changes is better we can show that the improvement is not significant. Significance is tested using a standard likelihood ratio test between the two models, comparing twice the difference in log-likelihood with a χ15672
 MathType@MTEF@5@5@+=feaafiart1ev1aaatCvAUfKttLearuWrP9MDH5MBPbIqV92AaeXatLxBI9gBaebbnrfifHhDYfgasaacH8akY=wiFfYdH8Gipec8Eeeu0xXdbba9frFj0=OqFfea0dXdd9vqai=hGuQ8kuc9pgc9s8qqaq=dirpe0xb9q8qiLsFr0=vr0=vr0dc8meaabaqaciaacaGaaeqabaqabeGadaaakeaaiiGacqWFhpWydaqhaaWcbaGaeyymaeJaeyynauJaeyOnayJaey4naCdabaGaeyOmaidaaaaa@335D@ distribution, where 1567 is the degrees of freedom by which the two models differ. Using the normal approximation for χ(1567,0.01)2
 MathType@MTEF@5@5@+=feaafiart1ev1aaatCvAUfKttLearuWrP9MDH5MBPbIqV92AaeXatLxBI9gBaebbnrfifHhDYfgasaacH8akY=wiFfYdH8Gipec8Eeeu0xXdbba9frFj0=OqFfea0dXdd9vqai=hGuQ8kuc9pgc9s8qqaq=dirpe0xb9q8qiLsFr0=vr0=vr0dc8meaabaqaciaacaGaaeqabaqabeGadaaakeaaiiGacqWFhpWydaqhaaWcbaGaeyikaGIaeyymaeJaeyynauJaeyOnayJaey4naCJaeyilaWIaeyimaaJaeyOla4IaeyimaaJaeyymaeJaeyykaKcabaGaeyOmaidaaaaa@39A9@ we compare (2(ln *L*_*general *_- ln *L*_*restricted*_)-1567)/2×1567
 MathType@MTEF@5@5@+=feaafiart1ev1aaatCvAUfKttLearuWrP9MDH5MBPbIqV92AaeXatLxBI9gBaebbnrfifHhDYfgasaacH8akY=wiFfYdH8Gipec8Eeeu0xXdbba9frFj0=OqFfea0dXdd9vqai=hGuQ8kuc9pgc9s8qqaq=dirpe0xb9q8qiLsFr0=vr0=vr0dc8meaabaqaciaacaGaaeqabaqabeGadaaakeaadaGcaaqaaiabikdaYiabgEna0kabigdaXiabiwda1iabiAda2iabiEda3aWcbeaaaaa@33AE@ = -9.71 with the relevant 99% critical value of 2.33 taken from a standard normal N
 MathType@MTEF@5@5@+=feaafiart1ev1aaatCvAUfKttLearuWrP9MDH5MBPbIqV92AaeXatLxBI9gBamrtHrhAL1wy0L2yHvtyaeHbnfgDOvwBHrxAJfwnaebbnrfifHhDYfgasaacH8akY=wiFfYdH8Gipec8Eeeu0xXdbba9frFj0=OqFfea0dXdd9vqai=hGuQ8kuc9pgc9s8qqaq=dirpe0xb9q8qiLsFr0=vr0=vr0dc8meaabaqaciaacaGaaeqabaWaaeGaeaaakeaaimaacqWFneVtaaa@383B@ (0,1). The difference is seen to be insignificant; the P-value is almost 1.

### Predicting protein secondary structure

We compared xrate to the phylo-HMM for prediction of protein secondary structure developed by Goldman, Thorne, and Jones [26] (here referred to as GTJ). This section uses a fully-connected three-state phylo-HMM with general reversible Markov chains. Training sets were taken from the HOMSTRAD database of structural alignments of homologous protein families [61].

We trained the phylo-HMM on alpha-beta barrel alignments from HOMSTRAD, leaving out the beta-glycanase SCOP family. xrate was then benchmarked on this beta-glycanase SCOP family to compare the annotation predicted by xrate to the experimentally determined HOMSTRAD annotation. We also tried a more comprehensive training regime, training xrate on the complete HOMSTRAD database (excluding the beta-glycanase SCOP family) and again comparing predicted and database annotations.

The performance of xrate was compared to that of GTJ. The results show that xrate can be used to quickly prototype and train a phylo-HMM with comparable performance to that reported by Goldman *et al*.

#### Grammar

**Figure 4:**
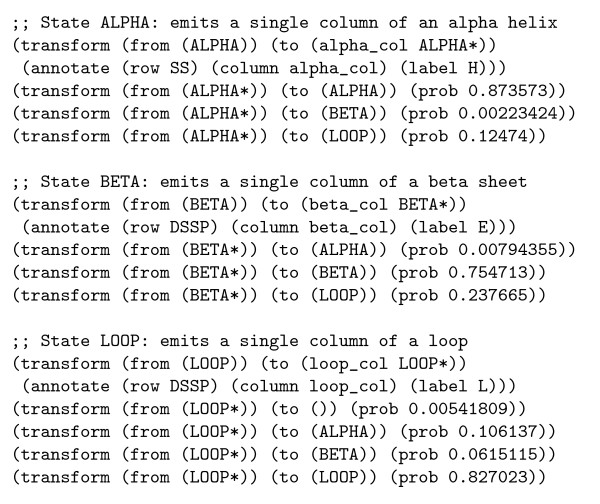
An excerpt from an xgram-format grammar reproducing the protein secondary structure phylo-HMM of Goldman, Thorne and Jones. This excerpt shows only the transformation rules, and omits the alphabet and chain definitions. Three separate Markov chains for amino acid substitution are used (and are assumed to be defined elsewhere in the file): alpha_col denotes an amino acid in an alpha helix (annotated with character H), beta_col denotes an amino acid in a beta sheet (annotated with character E) and loop_col denotes an amino acid in a loop region (annotated with character L).

The PROT3 phylo-grammar has state labels for the three secondary structure classes of alpha-helix (H), beta-sheet (E) and loop (L). An excerpt of the grammar is shown (see figure 4).

**Figure 5:**
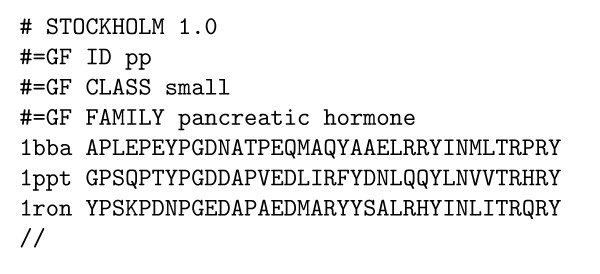
Example Stockholm-format input file for the protein secondary structure grammar (see figure 4). The alignment is of the pancreatic hormone family.

An example of usage for this grammar follows. We also show an alignment from HOMSTRAD, too small to predict secondary structure with any confidence, but useful for illustrative purposes (see figure 5). Suppose we want to: (1) read in this alignment from a file named ' pp. stk'; (2) load a point substitution matrix from a file named 'dart/data/nullprot.eg' (this is an amino-acid matrix distributed with xrate; the filename path assumes that the DART package was downloaded to the current working directory); (3) use the above point substitution matrix to estimate a phylo-genetic tree (by neighbor-joining followed by EM on the branch lengths); (4) load the PROT3 model from a file named 'dart/data/prot3.eg' (again, this is distributed with xrate); and (5) use the PROT3 model to predict secondary structure classes for this protein family, printing the annotated alignment to the standard output. The following command-line syntax achieves this:

xrate pp.stk --tree dart/data/nullprot.eg --grammar dart/data/prot3.eg

**Figure 6:**
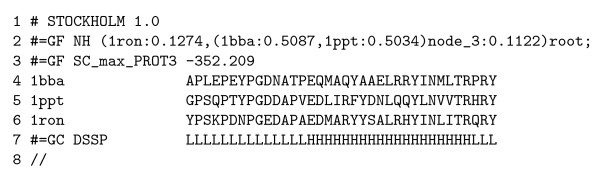
Example Stockholm-format output using the protein secondary structure grammar (see figure 4) and the pancreatic hormone alignment (see figure 5). Line numbers have been added for reference; note the embedded New Hampshire-format tree at line 2, the Viterbi bit-score at line 3 and the Viterbi secondary structure annotation at line 7.

The output of this command is shown (see figure 6).

**Figure 7:**
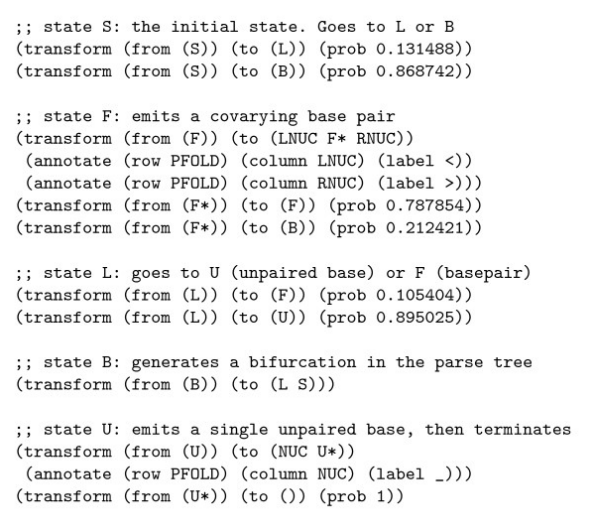
An excerpt from an xgram-format grammar reproducing the RNA secondary structure phylo-SCFG of Knudsen and Hein. This excerpt shows only the transformation rules, and omits the alphabet and chain definitions. Two separate Markov chains for nucleotide substitution are used (and are assumed to be defined elsewhere in the file): LNUC and RNUC denote the left and right (i.e. 5' and 3') nucleotides of a co-evolving basepair in a 16-state Markov chain (annotated with characters < and >), while NUC denotes an unpaired nucleotide in a 4-state Markov chain (annotated with character _).

**Figure 8:**
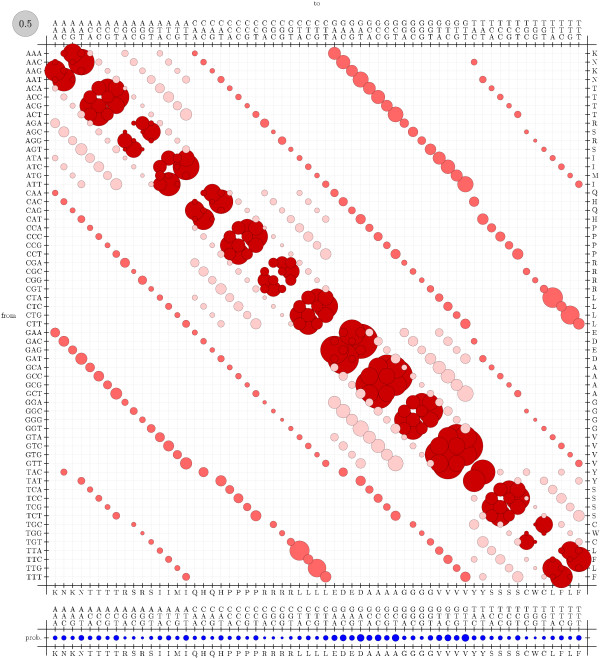
True codon mutation rate matrix for the M0 mechanistic codon mutation model benchmark (see Results and Discussion). These rates were used to generate simulated data; rates were then estimated from these data and compared to the true rates (see figure 9).

**Figure 9:**
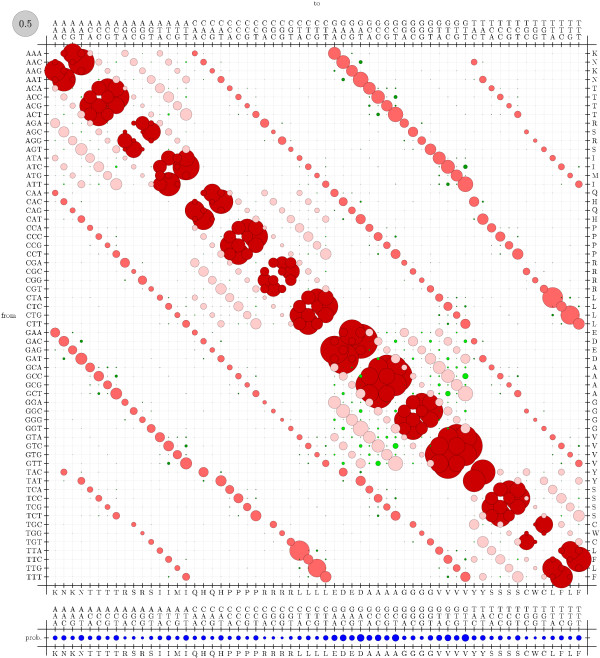
Estimated codon mutation rate matrix for the codon model benchmark (see Results and Discussion). These rates were estimated by xrate from simulated data, generated using a mechanistic rate model (see figure 8).

**Figure 10:**
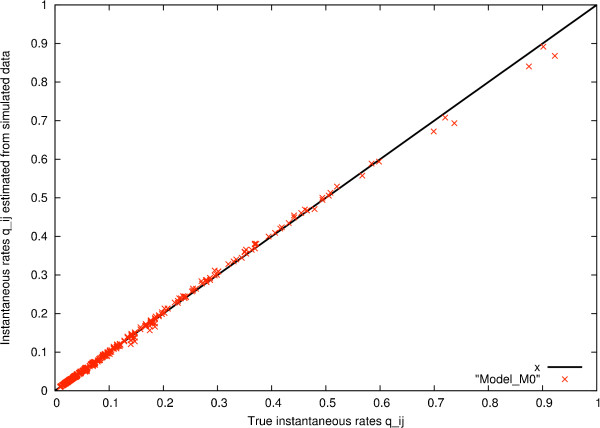
Scatter plot comparing true instantaneous rates with estimated rates from simulated data for the codon model benchmark (see Results and Discussion).

**Figure 11:**
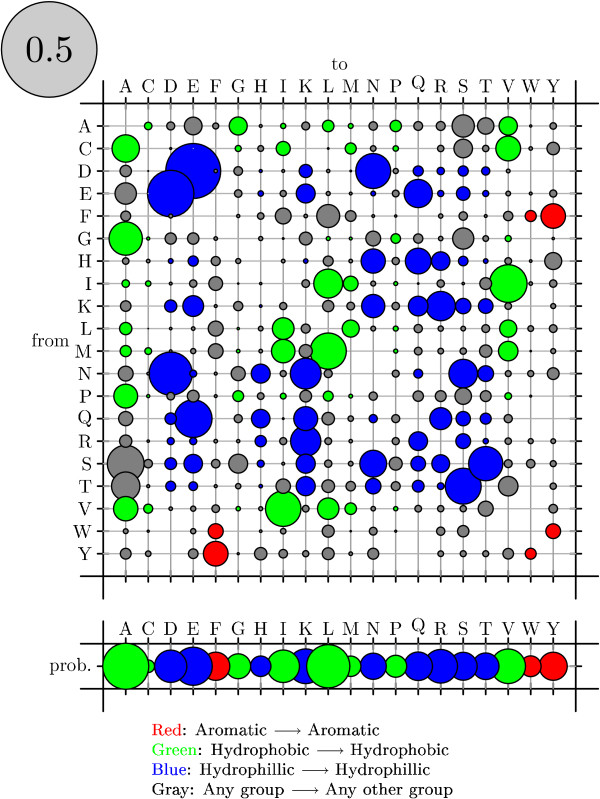
Bubbleplot of amino acid substitution rates for alpha-helices. See Results and Discussion for color-coding and explanation of bubbleplots.

More such examples can be found in DART (the software library with which xrate is distributed) and on the wiki pages for the xrate program [89]. A full list of command-line options for xrate can be obtained by typing xrate –help or, equivalently, xrate -h.

#### Results

Both xrate and the GTJ program were evaluated on the xylanase alignment used by GTJ, hereafter referred to as gtjxyl. xrate was trained on the subset of HOMSTRAD corresponding to alpha-beta barrel structures, with members of the beta-glycanase SCOP family (which includes the gtjxyl proteins) removed to prevent overlap between the training and test sets.

We report the prediction *accuracy *collectively for all secondary structure categories, and the *sensitivity *and *specificity *with respect to each individual category. These metrics are defined as follows

Sensitivity(*n*) = TP_*n*_/(TP_*n *_+ FN_*n*_)

Speciflcity(*n*) = TP_*n*_/(TP_*n *_+ FP_*n*_)

Accuracy = (∑nTPn
 MathType@MTEF@5@5@+=feaafiart1ev1aaatCvAUfKttLearuWrP9MDH5MBPbIqV92AaeXatLxBI9gBaebbnrfifHhDYfgasaacH8akY=wiFfYdH8Gipec8Eeeu0xXdbba9frFj0=OqFfea0dXdd9vqai=hGuQ8kuc9pgc9s8qqaq=dirpe0xb9q8qiLsFr0=vr0=vr0dc8meaabaqaciaacaGaaeqabaqabeGadaaakeaadaaeqbqaaiabbsfaujabbcfaqnaaBaaaleaacqWGUbGBaeqaaaqaaiabd6gaUbqab0GaeyyeIuoaaaa@3410@)/(∑nTPn
 MathType@MTEF@5@5@+=feaafiart1ev1aaatCvAUfKttLearuWrP9MDH5MBPbIqV92AaeXatLxBI9gBaebbnrfifHhDYfgasaacH8akY=wiFfYdH8Gipec8Eeeu0xXdbba9frFj0=OqFfea0dXdd9vqai=hGuQ8kuc9pgc9s8qqaq=dirpe0xb9q8qiLsFr0=vr0=vr0dc8meaabaqaciaacaGaaeqabaqabeGadaaakeaadaaeqbqaaiabbsfaujabbcfaqnaaBaaaleaacqWGUbGBaeqaaaqaaiabd6gaUbqab0GaeyyeIuoaaaa@3410@ + FN_*n*_)

where (for secondary structure class *n*) TP_*n *_is the number of true positives (columns correctly predicted as class *n*), FN_*n *_is the number of false negatives (columns that should have been predicted as class *n *but were not) and FP_*n *_is the number of false positives (columns that were incorrectly predicted as class *n*).

Bubbleplots were used to visualize the amino acid substitution rates. Substitutions are colored red if between aromatic amino acids, green if between hydrophobics and blue if between hydrophilics. Substitutions from one such group to another (e.g. from hydrophobic to hydrophilic) are colored gray.

**Figure 12:**
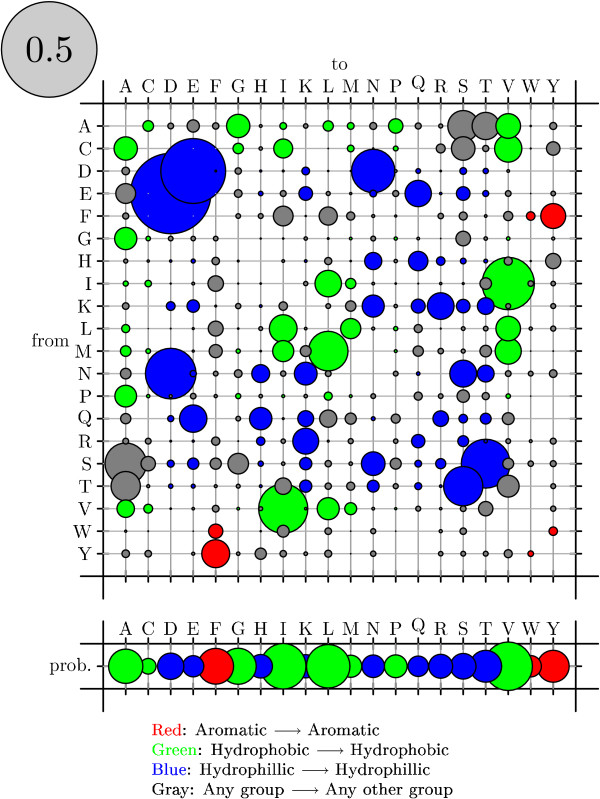
Bubbleplot of amino acid substitution rates for beta-sheets. See Results and Discussion for color-coding and explanation of bubbleplots.

**Figure 13:**
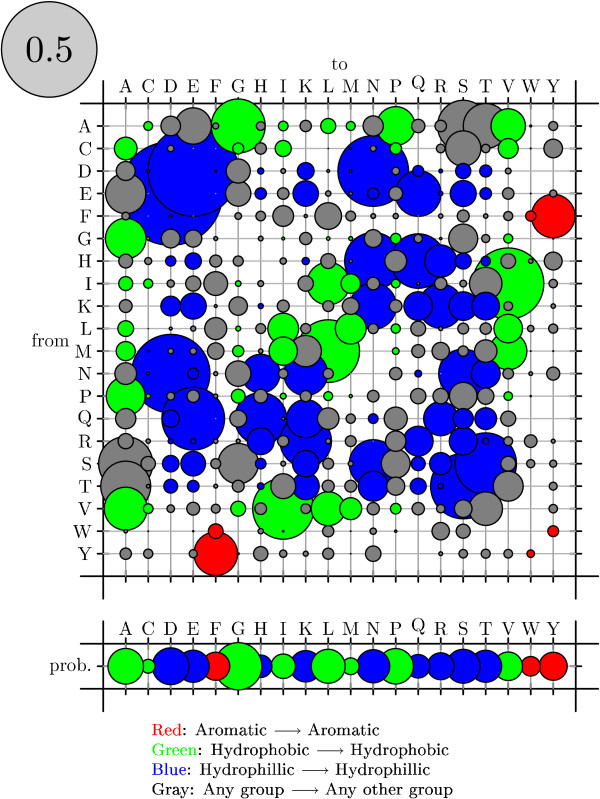
Bubbleplot of amino acid substitution rates for loop regions. See Results and Discussion for color-coding and explanation of bubbleplots.

Figures 11, 12 and 13 show the amino acid substitution matrices for the alpha-helix, beta-sheet and loop states, respectively. The relative rates displayed in the figures in general agree with what one would expect from each of those states: the alpha-helix and beta-sheet states substitute more slowly (and thus amino acid conservation is higher) than for the loop states (loop regions being more variable in structure [7]).

Table 1 shows the log likelihood scores of the training alignments, log *P*(*D*|*θ*), along with the log-posterior probability of the HOMSTRAD reference annotation, log *P*(*A*|*D*, *θ*). In this case, maximum-likelihood training also yields an increase in the annotation posterior probability *P*(*A*|*D*, *θ*). This is not in general a guaranteed result of the EM algorithm, and alternative training procedures (such as maximum-discrimination training [19]) have been proposed to achieve this effect. It appears in this case that such procedures are not required.

**Table 1:** Log-likelihood scores of training sets and log-posterior probabilities of the true annotations for the PROT3 benchmark. Here *D *denotes the training alignment data (the HOMSTRAD database without the beta-glycanase SCOP family), *A *denotes the DSSP annotations of the alignment data, *θ*_*D *_denotes the model with parameters obtained from training on *D*, and *θ*_*G *_denotes the model with parameters obtained from the GTJ datafiles.

*θ*	log_2 _*P*(*A*, *D*|*θ*)	log_2 _*P*(*D*|*θ*)	log_2 _*P*(*A*|*D*, *θ*)
*θ*_*D*_	-173038	-162491	-10547
*θ*_*G*_	-238632	-227979	-10653

Table 2 reports likelihoods, accuracies and runtimes for training set 2 as the EM convergence criteria are tightened. As expected, the likelihood increases as the convergence criteria are made more stringent. The annotation accuracy for the gtjxyl benchmark alignment also consistently increases.

**Table 2:** Effect of tightening the EM convergence criteria for the PROT3 benchmark. The "mininc" parameter is the minimum fractional log-likelihood increase per iteration of EM. Accuracies for the gtjxyl benchmark alignment are reported, along with log-likelihoods. See Table 1 for additional notation.

mininc	Runtime/min	Acc(gtjxyl)	log_2 _*P*(*A*, *D*|*θ*_*D*_)	log_2 _*P*(*D*|*θ*_*D*_)	log_2 _*P*(*A*|*D*, *θ*_*D*_)
le-3	14	64.1	-2696469	-2549947	-146522
le-4	35	64.7	-2686598	-2539908	-146690
le-5	84	68.0	-2682667	-2536849	-145818

Table 3 summarizes the results of running xrate and the GTJ program on all the test cases. In general the accuracy of xrate is comparable to or even slightly better than the accuracy of the GTJ program.

**Table 3:** Summary of prediction performance for the PROT3 benchmark. "Sn" and "Sp" are the sensitivity and specificity for each secondary structure category; "Acc" is the overall accuracy.

Program	Sn (*α*)	Sp (*α*)	Sn (*β*)	Sp (*β*)	Sn (L)	Sp (L)	Acc
GTJ	66.7	91.3	63.5	84.0	73.5	77.3	69.6
xrate	71.6	95.7	82.7	79.0	65.2	81.2	70.2

### Predicting RNA secondary structure

To illustrate the capability of xrate as a tool for RNA secondary structure prediction/annotation, we compare it to Pfold, a phylo-SCFG developed by Knudsen and Hein [46,47].

There are two goals of this section: (1) to see if xrate can exactly emulate the Pfold phylo-grammar using the same parameters as Pfold, and (2) to see if the EM algorithm can estimate parameters that yield comparable performance to those produced by other methods.

We benchmarked the Pfold phylo-SCFG running on xrate against the original Pfold program using alignments from the Rfam database [30]. To address goal (2), we used xrate to estimate the substitution rates and initial frequencies of basepairs and single nucleotides from annotated Rfam alignments.

Our results show that the Pfold phylo-SCFG is effectively emulated by xrate, that the EM algorithm can estimate a more likely parameterization for a given training set and that the parameters so obtained are comparable in performance to the Pfold program itself. We conclude that xrate is a suitable platform for developing, parameterizing, and testing phylo-grammars without the necessity of writing source code or performing manual parameterization.

#### Grammar

The PFOLD grammar is taken from the Pfold program and is described in the paper by Knudsen and Hein [46].

An excerpt of the grammar, containing the production rules, is seen in figure 7 . 

#### Results

We report the *sensitivity *and *positive predictive value *(PPV) of basepair predictions. These accuracy metrics are defined as follows

Sensitivity = TP/(TP + FN)

PPV = TP/(TP + FP)

where TP is the number of true positives (base pairs that are predicted correctly per the Rfam annotation), FN the number of false negatives (base pairs that are not predicted but are in the Rfam annotation) and FP the false positives (predicted base pairs that are not in the Rfam annotation).

Training and testing sets were obtained by selecting the 148 RNA gene families in Rfam version 7 with experimentally-determined structures, discarding pseudoknots, removing excessively gappy columns (as this step is also performed by Pfold), grouping the families into superfamilies and randomly partitioning these superfamilies into two sets [see Additional file 1]. This yielded a training set of 71 alignments and a testing set of 77 alignments.

The benchmark results, shown in Table 4, indicate that the sensitivity and PPV of the Pfold program and its emulation on xrate are comparable. It should be noted, however, that the sets of base pairs predicted by the two programs are slightly different [see Additional file 1]. After examination, we attribute this to differences in implementation and loss of precision due to numerical calculations.

**Table 4:** Accuracy of RNA secondary structure prediction. Comparison of sensitivities and PPVs for the Pfold program, its phylo-SCFG running on xrate with its original rates, and its phylo-SCFG running on xrate with rates estimated from Rfam by the phylo-EM algorithm.

	Sensitivity	PPV
Pfold	45.0%	58.3%
xrate emulating Pfold	44.4%	61.7%
xrate trained on Rfam	42.8%	58.2%

**Figure 14:**
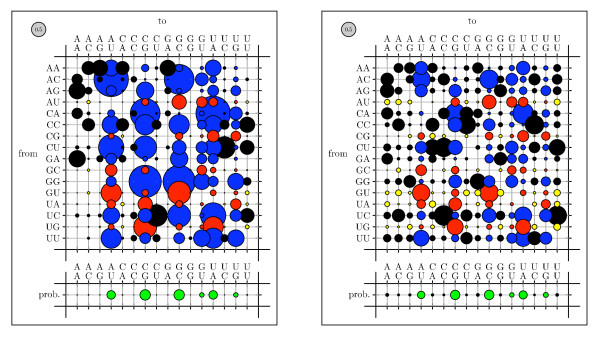
Comparison of basepair substitution rates, colored by basepairing conservation, gain, or loss. Rates and equilibrium frequencies from the Pfold phylo-SCFG (left panel) are compared with those estimated by the phylo-EM algorithm from Rfam (right panel). Substitutions from non-canonical to canonical basepairs are blue (pairing gain), canonical to canonical are red (pairing conservation), non-canonical to non-canonical are black (unpaired and no change), and canonical to non-canonical are yellow (pairing loss).

**Figure 15:**
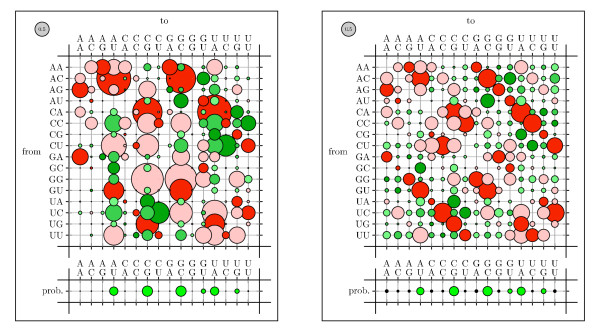
Comparison of basepair substitution rates, colored by transitions/transversions. The rates were obtained from the Pfold program and by training on Rfam (see figure 14). Transition of a single base in a pair is dark red, transversion is light red; transitions in both bases is dark green, transition of one and transversion of the other is medium green, transversions of both is light green.

**Figure 16:**
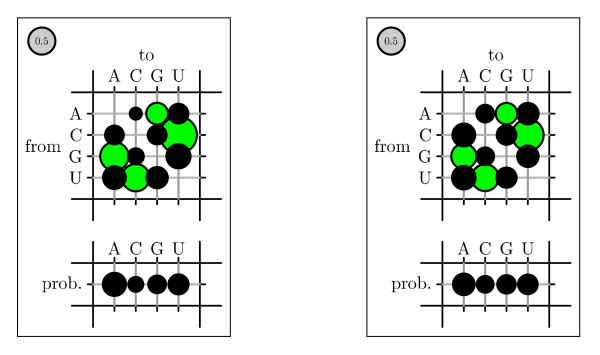
Comparison of substitution rates of nucleotides in unpaired alignment columns. Rates and equilibrium frequencies from the Pfold phylo-SCFG (left panel) are compared with those estimated by the phylo-EM algorithm from Rfam (right panel). Transitions are green, transversions are black.

We also tested whether parameterizing the phylo-SCFG using the EM algorithm is comparable to the Pfold parameterization [46]. A comparison of Pfold's original rates with the EM-estimated rates is shown in Figures 14–16. Both sets of parameters display similar trends. Substitutions that create or preserve canonical base pairs are more frequent than substitutions that destroy basepairs (see figure 14). Transitions are more common than transversions, both within basepairs (see figure 15) and unpaired sites (see figure 16). There is a difference in the magnitude of many of the rates, which we attribute to differences in the training sets.

The predictive accuracy of Pfold is compared to that of the xrate-trained phylo-SCFG in Table 4, while log-likelihoods are compared in Tables 5 and 6. The results are similar, indicating that the combination of training set and xrate-implemented EM is comparable to the training procedure used in the development of Pfold.

**Table 5:** Log-likelihoods of alignments, and log-posteriors of alignment annotations, for training and testing datasets under various EM convergence regimes in the PFOLD benchmark. The "mininc" parameter is the minimal fractional increase in the log-likelihood that is considered by our EM implementation to be an improvement, while the "forgive" parameter is the number of iterations of EM without such an improvement that will be tolerated before the algorithm terminates. The default settings are mininc = le-3, forgive = 0. Here *D *denotes the alignment data, *A *denotes the RFAM secondary structure annotations of the alignment data and *θ *denotes the model with parameters optimized for the training set using the specified EM convergence criteria.

Dataset	"mininc"	"forgive"	log_2 _*P*(*D*, *A*|*θ*)	log_2 _*P*(*D*|*θ*)	log_2 _*P*(*A*|*D*, *θ*)
Training set	le-3	0	-466330.6649	-453589.9251	-12740.7398
Training set	le-4	0	-465397.0642	-453403.7081	-11993.3561
Training set	le-5	0	-465397.0642	-453403.7081	-11993.3561
Training set	le-3	2	-465821.5239	-453476.0389	-12345.4850
Training set	le-3	4	-465565.9224	-453437.5353	-12128.3871
Training set	le-3	6	-465397.0642	-453403.7081	-11993.3561
Training set	le-3	8	-465291.1983	-453356.6841	-11934.5142
Training set	le-4	4	-465147.9174	-453318.4543	-11829.4631
Training set	le-4	10	-465010.8431	-453209.0744	-11801.7687
Test set	le-3	0	-360472.7960	-343832.6014	-16640.1946
Test set	le-4	0	-360190.7940	-344117.5123	-16073.2817
Test set	le-5	0	-360190.7940	-344117.5123	-16073.2817
Test set	le-3	2	-360148.9090	-343841.2775	-16307.6315
Test set	le-3	4	-360178.4500	-344016.2558	-16162.1942
Test set	le-3	6	-360190.7940	-344117.5123	-16073.2817
Test set	le-3	8	-360092.2930	-344078.8868	-16013.4062
Test set	le-4	4	-360057.4880	-344116.5923	-15940.8957
Test set	le-4	10	-360108.0100	-344166.2108	-15941.7992

**Table 6:** Log-likelihoods of alignments, and log-posteriors of alignment annotations, for training and testing datasets using the original Pfold program. Comparison with Table 5 shows that EM training increases all probabilities, as desired.

Dataset	log_2 _*P*(*D*, *A*|*θ*)	log_2 _*P*(*D*|*θ*)	log_2 _*P*(*A*|*D*, *θ*)
Training set	-487422.5964	-464828.9148	-22593.6816
Test set	-370490.5284	-348550.7516	-21939.7768

**Figure 17:**
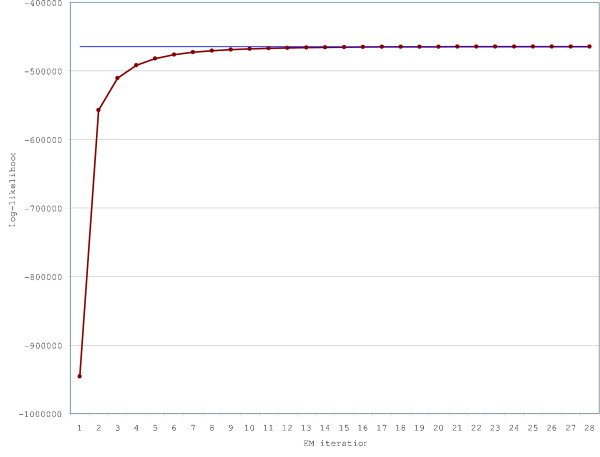
Log-likelihoods (log2 P(alignment, annotation|parameters), red line) increase as the EM algorithm optimizes the model parameters on the training set. The accuracy results for this parameterization are reported in Table 4. The blue line represents the asymptotic best log-likelihood, reached at iteration 27.

An important point to check is whether the EM algorithm actually performs as designed. We expect to see certain phenomena if the algorithm is indeed working as expected:

• The algorithm, over the course of its iterations, should refine the parameter set (denoted at the *n*'th iteration by *θ*^(*n*)^) to maximize the likelihood of the alignment data *D *and (if supplied) the annotation *A*. Therefore, the log-likelihood log *P*(*D*|*θ*^(*n*)^) should increase with *n *towards an asymptotic maximum value. This is indeed observed to be the case for this example (see figure 17).

• In practice, the EM algorithm is not run for an infinite number of iterations; rather, the algorithm stops when some "convergence criteria" are met (relating to the fractional increase of the log-likelihood) and the parameters at this point are considered to be the "convergent parameters". We denote this convergent parameter set by θ*.

• If the EM algorithm is performing effectively (i.e. finding a parameterization whose likelihood is close to the global maximum), we would also expect *P*(*D*|θ*) to be greater than *P*(*D*|*θ*') for some arbitrarily chosen parameterization *θ*' (for example, the Knudsen-Hein parameters, which were optimized for a dataset other than *D*). A comparison of Tables 5 and 6 confirms this to be the case.

• As the convergence criteria become more strict, log *P*(*D*|θ*) should increase. The results in Table 5 confirm this to be the case.

• If the training set is representative of the test set, then the above statements should also hold true when *D *is taken to mean the test set. Again, Tables 5 and 6 confirms this.

We note that Tables 5 and 6 shows that the posterior probability of the true annotation, *P*(*A*|*D*, *θ*) = *P*(*A*, *D*|*θ*)/*P*(*A*|*θ*), is also increased after phylo-EM training. As mentioned above, this is not a provably guaranteed result of the EM algorithm, which is designed to maximize only *P*(*A*, *D*|*θ*).

## Conclusion

We have developed a tool, xrate, that combines the power of stochastic grammars, phylogenetic models, and fast automated parameter estimation from training data. The tool combines a novel EM algorithm for estimating rate parameters of the general irreversible substitution model (extending our earlier results for reversible models [38]) with the Forward-Backward and Inside-Outside algorithms familiar from the stochastic grammar literature [16]. Novel grammars can be designed by the user, trained automatically, and evaluated without the need for writing or compiling any code. Example grammars that we have used with xrate so far include the phylo-HMMs used by Thorne, Goldman and Jones to predict protein secondary structure [83], the phylo-SCFGs used by Knudsen and Hein to predict ncRNA structure [46] and the DNA phylo-HMMs used by Siepel and Haussler to predict protein-coding genes and find highly-conserved elements [81,80,39,79].

There are many useful applications of stochastic grammars in bioinformatics. Past triumphs of HMMs include protein homology detection [49]; prediction of protein-coding genes [10]; transmembrane and signal peptide annotation [42]; and profiles of fragment libraries for *de novo *protein structure prediction [76]. Applications of "higher-power" stochastic grammars (i.e. grammars that are situated further up the Chomsky hierarchy, such as Tree-Adjoining Grammars [40]) include beta-sheet prediction [1]; RNA genefinding [74], homology detection [17] and structure prediction [73]; and operon prediction [6].

There are also many useful applications of phylogenetic models. These include reconstruction of phylogenetic trees [22], measurement of *K*_*a*_/*K*_*s *_ratios [27], modeling residue usage [9,31], modeling covariation [71], detecting of conserved residues [90] and sequence alignment [84,33,37]. Furthermore, there are many applications of probabilistic modeling in sequence analysis, e.g. "evolutionary trace" [52] or prediction of deleterious SNPs [65], that are either directly related to the above kinds of models or might productively be linked.

xrate and associated tools comprise an up-to-date, friendly implementation of these models for the advanced user. We believe these are powerful tools with broad utility. Our results show that the performance of xrate is comparable to previously described phylo-HMM and phylo-SCFG implementations customized to specific tasks, and furthermore that the rate estimates produced by xrate can be interpreted in a biologically meaningful way. In releasing this general implementation, our hope is that we and others will use these computational tools to further the application of molecular evolution in biomedical research.

## Availability and requirements

**Project name **: xrate

**Project home page **: 

**Operating system(s) **: Platform independent

**Programming language **: C++

**Other requirements **: gcc version 3.3 or higher; GNU build tools (make, ar)

**License **: GNU GPL

**Restrictions to use **: None

## Abbreviations

**CYK **: Cocke-Younger-Kasami

**DP **: Dynamic Programming

**EM **: Expectation Maximization

**HMM **: Hidden Markov Model

**ML **: Maximum Likelihood

**PPV **: Positive Predictive Value

**SCFG **: Stochastic Context-Free Grammar

## Authors' contributions

PK implemented the irreversible phylo-EM algorithm and contributed to the supplementary material describing the algorithm. NG and RB developed the bubbleplot code. CK and NG performed the codon benchmark. YB performed the protein secondary structure benchmark. AU performed the RNA secondary structure. RB and SC performed additional benchmarks and testing of xrate. IH developed the remaining code and drafted the manuscript. IH, CK, NG, YB and AU contributed to the final version of the manuscript.

## Supplementary Material

Additional File 1 XRate: a fast prototyping, training and annotation tool for phylo-grammars. Supplementary material. A full description of the phylo-EM algorithm for irreversible substitution models. Also contains details of experimental procedures. Click here for file
