# Supplementary material for: XRate: a fast prototyping, training and annotation tool for phylo-grammars
Source: BMC Bioinformatics. 2006 Oct 3;7:428. doi: 10.1186/1471-2105-7-428 (PMC1622757; doi:10.1186/1471-2105-7-428)
Supplement: Additional File 1 — XRate: a fast prototyping, training and annotation tool for phylo-grammars. Supplementary material. A full description of the phylo-EM algorithm for irreversible substitution models. Also contains details of experimental procedures. [file 1471-2105-7-428-S1.pdf]

XRate: a fast prototyping, training and annotation tool for phylo-grammars
Supplementary material

P.S.Klosterman, I.H.Holmes et al
Department of Bioengineering
University of California, Berkeley

1 Rate and probability functions

Formally, we construct the sets of allowable rate and probability functions in an xgram grammar file as follows. Suppose that λ denotes any rate parameter, p any probability parameter, k a nonnegative real constant and $\theta = \{\lambda, p, \ldots\}$ the set of all rate and/or probability parameters. Then the set of allowable probability functions is constructed as follows:

- $f(\theta) = k$ is an allowable probability function;
- $f(\theta) = p$ is an allowable probability function;
- If $f(\theta)$ and $g(\theta)$ are allowable probability functions, then $h(\theta) = f(\theta)g(\theta)$ is an allowable probability function;
- If $f(\theta)$ and $g(\theta)$ are allowable probability functions, then $h(\theta) = f(\theta) + g(\theta)$ is an allowable probability function.

The set of allowable rate functions is constructed as follows:

- $f(\theta) = \lambda$ is an allowable rate function;
- If $f(\theta)$ is an allowable rate function and $g(\theta)$ is an allowable probability function, then $h(\theta) = f(\theta)g(\theta)$ is an allowable rate function;
- If $f(\theta)$ and $g(\theta)$ are allowable rate functions, then $h(\theta) = f(\theta) + g(\theta)$ is an allowable rate function.

Allowable probability functions may be used in place of probability-like constants in the grammar (i.e. probabilities for grammar rules & initial state occupancies for rate matrices). Allowable rate functions may be used in place of rate-like constants in the grammar (i.e. substitution rates).

2 Experimental methods

2.1 The M0 codon model

The parameters used for the M0 mechanistic model of codon evolution were ω= 0.2 and κ = 2.5.

2.2 The PROT3 grammar

The HOMSTRAD database is grouped into ten secondary structure classes and contains no two sequences with greater than 90% sequence identity. The 1032 alignments in the HOMSTRAD database were converted to Stockholm format. The DSSP secondary structure annotation was converted into a consensus annotation containing the three secondary structure states using the same mapping as described in the GTJ paper. The consensus was determined by majority; ie, the annotation within a column with the greatest frequency was declared the consensus.

The GTJ program and associated files were downloaded from the GTJ FTP site. The GTJ program and `xrate` were both evaluated on the xylanase alignment, here referred to as gtjxyl. In addition they were both evaluated on the "glycosyl hydrolases family 10" (referred to here as ghf10) alignment from HOMSTRAD (data not shown). This alignment is in the alpha-beta barrel class and includes the endo-1,4-beta-xylanase A protein from *P. fluorescens*. This alignment is very similar to the psefl alignment and so was used as an additional test of the performance of the GTJ method and `xrate`. The ghf10 alignment is part of the beta-glycanase SCOP family which also includes the ghf5 and ghf17 alignments in HOMSTRAD. All three alignments were removed when creating the training databases. Furthermore, the training databases were queried using BLAST to ensure that no homologs to the test sequences were found.

In order to train on the databases, `xrate` was first used to estimate a phylogenetic tree for each alignment. These trees were estimated by neighbor-joining followed by EM to optimize the branch lengths. A point substitution rate matrix, estimated from a 200-family subset of PFAM, was used for this step. This point substitution matrix was also used as the "seed" for training the rate matrices of the PROT3 grammar.

For most of the EM training, the "mininc" parameter was set to the default value of 0.001, so that for each iteration of EM, the fractional increment in the log-likelihood was required to be at minimum 0.001 if `xrate` was to regard the increase as an improvement. The "forgive" parameter was set to 5, meaning that `xrate` would allow five iterations without such an improvement before terminating.

The exact training set used by GTJ was not available, but a closely similar dataset was used for the purposes of comparison.

The model parameters used by the GTJ program were converted to phylo-grammar format. The substitution rates were scaled so that the average substitution rate for the loop state is 1, as per GTJ. The secondary structures predicted by `xrate` and the GTJ program were compared to the annotated "true" secondary structure. Statistics were calculated for the sensitivity and specificity of the predictions for the three secondary structure states as well as overall prediction accuracy.

2.3 The PFOLD grammar

We attempted to replicate the Pfold program using `xrate` by inputting the Pfold phylo-grammar (which consists of the SCFG, its production probabilities, the initial frequencies and substitution rates of base pairs and unpaired nucleotides, and a parameter denoting that the model is reversible) to `xrate` as a file in the S-expression format, then using `xrate` with this grammar to perform secondary structure annotation on a testing set containing multiple sequence alignments from Rfam. The results were compared with annotation done by the Pfold program (2003 version).

The annotation of the data by a stochastic grammar is often determined from the most optimal parse of the data according to the grammar. In the case of SCFGs, this parse is computed by the CYK algorithm. However, Pfold uses posterior decoding theory to find the annotation containing the highest expected number of correct predictions, summed over all parses. Our benchmarks indicate that this approach produces a greater basepair PPV, but lower sensitivity, than the CYK approach (data not shown). To enable consistent testing, we modified Pfold to revert to outputting the secondary structure using CYK, as `xrate` does. Conceivably, `xrate` could be modified to produce an annotation in some manner other than CYK. However, due to the limitless variety of objective functions which can be used to do posterior decoding, we have opted not to implement any single posterior decoding method, but rather included an option to print out the entire table of column posterior probabilities in the `xrate` output. This allows such methods to be implemented as an additional layer on top of the `xrate` software.

For both Pfold and `xrate`, a phylogeny tree is required for each alignment showing the evolutionary relationship among the sequences. These trees and their branch lengths were estimated by `xrate` using

the Jukes-Cantor model. We then used these trees for both the Pfold and the `xrate` predictions. Pfold implements a slightly different tree construction method; we use this model here for simplicity and consistency between the two methods.

One final measure of data preparation must be done to make Pfold and `xrate` perform similarly enough for a benchmark: the difference in their handling of alignment columns containing gaps must be taken into account. Pfold employs a heuristic where columns containing gaps in 25% of the sequences or more are not considered for annotation, which generally increases PPV, but lowers sensitivity of base pair predictions. For consistency, we removed all such columns before showing the alignment data to `xrate` or Pfold.

The training set and testing set of multiple sequence alignments were prepared from Rfam version 7 as follows. 148 alignments of RNA families, including only experimentally-determined secondary structure annotations curated from published articles in the literature, were partitioned into 13 superfamilies according to the Rfam grouping: cis-reg (frameshift, IRES, riboswitch, thermoregulator, other) and gene (antisense, miRNA, ribozyme, rRNA, guide snRNA, splicing snRNA, sRNA, other). Note that tRNA and Group I catalytic intron alignments (Rfam IDs RF00005 and RF00028, respectively) were not part of these sets because they only had one alignment per superfamily. Alignments in each of the 13 superfamilies were randomly partitioned into two sets, with one added to the training set, the other added to the testing set. Pseudoknots, comprising a small fraction (175 of the 5780 base pairs) of the two sets, were removed from all annotations. Additionally, alignment RF00061 (containing 98 base pairs after pseudoknot removal) was removed from the test set because it was causing Pfold's implementation of the CYK algorithm to crash. Lastly, because Pfold's SCFG cannot generate single-nucleotide hairpin loops, the only instance of such a loop in the two sets – in RF00232 – was changed to a hairpin loop of 3 unpaired nucleotides by removing the annotation for the closing base pair.

The above procedure yielded a training set of 71 alignments and a testing set of 77 alignments.

Trees for each alignment/family in the training set and testing set were estimated with `xrate` using the Jukes-Cantor model of nucleotide substitution.

Various pseudocounts (for the EM update statistics) and EM convergence criteria were tried to prevent division-by-zero errors and to maximize accuracy of the Rfam-trained Pfold phylo-SCFG. Empirically, we found that adding a pseudocount of 0.001 to start times and mutation counts gave the best results.

As the EM algorithm requires an initial substitution rate matrix (or, as in this case, matrices) to improve upon over its iterations, a seed phylo-grammar must be created. For the seed, we chose rate matrices where the mutation rates are all equal but low, so that the total mutation rate from any state is $\ll 1$; empirically, we find that "low-balling" the seed like this improves the rate of convergence, as fewer spurious mutations are estimated in the initial rounds of EM.

`xrate` and Pfold predict slightly different basepair sets. `xrate` predicts 490 base pairs that Pfold does not, Pfold predicts 698 base pairs that `xrate` does not, and both predict 1487 same base pairs. The accuracy of the methods is nonetheless comparable.

3 The Phylo-EM algorithm

This appendix covers the spectral theory of continuous-time finite Markov chains (reversible and irreversible) for comparative sequence analysis, including calculations of the matrix exponential (using either Taylor series or Padé approximant with scaling and squaring, or eigenvector decomposition), Felsenstein's pruning and peeling algorithms for phylogenetic trees, and the Expectation Maximisation algorithm for maximum likelihood parameterisation.

3.1 Notation

Vectors are written in bold-face and lower-case ($\mathbf{p}$, $\mathbf{q}$) with single-subscripted elements in normal-face (p_i, q_j).

Matrices are written in bold-face and upper-case ($\mathbf{Q}$, $\mathbf{R}$) with double-subscripted elements in normal-face (Q_{ij}, R_{kl}).

3.2 The state space

In this section, "state" refers to a state of a continuous-time Markov chain, rather than a nonterminal symbol in a stochastic grammar (for which the term "state" is occasionally also used, particularly if the stochastic grammar is a Hidden Markov Model).

Suppose there are N states. (We assume for now that the state space is finite, though this can be relaxed. The state space must always, however, be discrete.) Let $\sigma(t)$ be the state of the system at time t.

Examples of finite state spaces include the set of all nucleotides at a given site in an RNA sequence ($N = 4$), the set of all dinucleotide pairs ($N = 4^2$), the set of all tetranucleotides ($N = 4^4$) or the set of all stems of length L ($N = 4^{2L}$).

Examples of infinite state spaces include the set of all nucleotide sequences and the set of all RNA secondary structures.

3.3 The rate matrix and the equation of state

Let Q_{ij} be the instantaneous rate of mutation from state i to state j, where $i \neq j$. Let $-Q_{ii} = \sum_{j \neq i} Q_{ij}$ be the *exit rate* from state i. Note that Q_{ii} is negative. The $N \times N$ matrix, $\mathbf{Q}$, is called the *rate matrix*.

Let $p_i(t) = P(\sigma(t) = i)$ be the probability that, at time t, the system is in state i. The N-element vector, $\mathbf{p}$, is called the *state vector*.

We will be right-multiplying $\mathbf{p}$ by $\mathbf{Q}$, forming the product $\mathbf{pQ}$ rather than the product $\mathbf{Qp}$. This is a result of the way we have chosen to order $\mathbf{Q}$ (the rate from i to j is Q_{ij} rather than Q_{ji}). Consequently we must treat $\mathbf{p}$ as a row vector rather than a column vector.

The *equation of state* for the continuous-time discrete-state Markov chain is

$$\frac{d}{dt}\mathbf{p} = \mathbf{pQ} \tag{1}$$

This is a matrix ODE (ordinary differential equation). Expanding this for the j'th state, we obtain $\frac{d}{dt}p_j = \sum_{i \neq j}(p_i Q_{ij} - p_j Q_{ji})$. The first term represents mutations entering state j, while the second term represents mutations leaving state j.

3.4 Discrete-time analogy

We can get some insight into the behaviour of the equation of state (1) if we consider breaking up the time axis into short discrete steps of size Δt. Let $\sigma(m)$ be the state at the m'th step, i.e. at time $T = m\Delta t$, and let $\mathbf{p}(m)$ be the state vector. The process $\sigma(m)$ is a discrete-time Markov chain, which may be more familiar as it is similar in some ways to the kind of Hidden Markov Model (HMM) that is used for bioinformatic sequence analysis (although in bioinformatics, the time variable actually means "position along the sequence").

If Δt is small, the probability of transiting from state i to a different state j in one time-step is $R_{ij} = Q_{ij}\Delta t$, while the probability of staying in state i is $R_{ii} = 1 - \sum_{j \neq i} Q_{ij}\Delta t = 1 + Q_{ii}\Delta t$. Thus, the discrete version of the equation of state is

$$\mathbf{p}(m+1) = \mathbf{p}(m)\mathbf{R}$$

with $\mathbf{R} = \mathbf{I} + \mathbf{Q}\Delta t$, where $\mathbf{I}$ is the identity matrix. Here $\mathbf{R}$ is the discrete-time transition probability matrix for short time intervals Δt.

(Note that $\mathbf{R} = \mathbf{I} + \mathbf{Q}\Delta t$ is the first-order Taylor series approximation to the true discrete-time transition matrix, which is $\exp(\mathbf{Q}\Delta t)$; see section 3.5. The approximation will be best for small Δt.)

The general solution to the discrete equation of state is $\mathbf{p}(m) = \mathbf{p}(0)\mathbf{R}^m$. Note that the continuous-time matrix exponential $\exp(\mathbf{Q}t)$ is replaced by $\mathbf{R}^m = (\mathbf{I} + \mathbf{Q}\Delta t)^m$ in the discrete case. An implication of this is that we can define the matrix exponential as the following continuous-time limit

$$\exp(\mathbf{Q}T) = \lim_{\Delta t \to 0} (\mathbf{I} + \mathbf{Q}\Delta t)^{T/\Delta t}$$

where we have used $m = T/\Delta t$. We will revisit this continuous-time limit later, in the context of parameter estimation.

3.5 The matrix exponential

By analogy with the corresponding scalar ODE, we can write the solution to the equation of state as $\mathbf{p}(t) = \mathbf{p}(0)\mathbf{M}$ where $\mathbf{M} = \exp(\mathbf{Q}t)$ is the *matrix exponential.*

This should not seem strange—since, for example, the scalar exponential $x = \exp(kt)$ is often (formally) defined as the solution to the equation $\frac{dx}{dt} = kx$ with boundary condition $x(0) = 1$. We have just done the same thing, but with a matrix $\mathbf{Q}$ instead of a scalar k. Of course, writing down a symbolic solution is one thing; in practise we have to figure out some means of computing the actual entries of $\mathbf{M}$.

One way of doing this is via the Taylor series. For scalars, this is the following infinite series.

$$x(t) = \exp(kt) = 1 + kt + \frac{1}{2}(kt)^2 + \frac{1}{6}(kt)^3 + \ldots = \sum_{n=0}^{\infty} \frac{1}{n!}(kt)^n$$

It can be verified that this satisfies the scalar ODE $\frac{dx}{dt} = kx$. Furthermore, this remains true when the scalars x, k are replaced with matrices $\mathbf{M}, \mathbf{Q}$.

Of course, we can't evaluate an infinite series in finite compute time. However, if kt (or $|\mathbf{Q}t|$) is small, then only the first few terms in the Taylor series will contribute much to the result. We can think of the n'th term, $\frac{1}{n!}(\mathbf{Q}t)^n$, as representing the effect of n successive mutations in the time interval $[0, t]$. If t is small compared to $1/\rho$, where ρ is the expected mutation rate, then only a few mutations are likely to have occurred. Thus, we can expect the Taylor series to converge at quite small n.

In the case where kt (or $|\mathbf{Q}t|$) is not small, we use the method of scaling and squaring, as described in Moler and Van Loan's Nineteen Dubious Ways to Compute the Exponential Matrix. This exploits a fundamental property of the exponential function: $e^A = (e^{A/m})^m$. m is chosen to be a power of two for which $e^{A/m}$ can be efficiently computed; $(e^{A/m})^m$ is computed by repeated squaring. In practice, the Padé approximant is more efficient than the Taylor series; Gerton Lunter has kindly provided an implementation of matrix exponentiation using the Padé approximant with scaling and squaring.

In general, each matrix multiplication (& hence each term of the series) takes compute time $O(N^3)$. However, if the rate matrix $\mathbf{Q}$ is sparse, this can be improved on.

3.6 Diagonalising the rate matrix

There is an exact way of evaluating the matrix exponential $\mathbf{M} = \exp(\mathbf{Q}t)$, by diagonalising the rate matrix. To do this we first need to find the eigenvalues and eigenvectors of $\mathbf{Q}$. This is a straightforward application of matrix algebra.

An eigenvalue and the associated (right) eigenvector of the matrix $\mathbf{Q}$ are a scalar μ and a column vector $\mathbf{u}$ satisfying $\mathbf{Qu} = \mu\mathbf{u}$. This implies that $(\mathbf{Q} - \mu\mathbf{I})\mathbf{u} = \mathbf{0}$ where $\mathbf{I}$ is the identity matrix. Therefore

$$|\mathbf{Q} - \mu\mathbf{I}| = 0$$

where $|\mathbf{X}|$ represents the determinant of matrix $\mathbf{X}$. This is the *characteristic equation.* In general the polynomial in this equation (the *characteristic polynomial*) has degree N, so there are N solutions and hence N eigenvalues (though some of these may be identical, or *degenerate*).

Once we have found an eigenvalue, by solving the characteristic equation or by other means, we can find the corresponding eigenvector. Let $\mathbf{D}$ be the diagonal matrix whose on-diagonal entries are the N eigenvalues, μ_k, and let $\mathbf{U}$ be the matrix whose columns are the corresponding (right) eigenvectors, $\mathbf{u}^{(k)}$. It follows that $\mathbf{QU} = \mathbf{UD}$.

We here consider diagonalizable matrices $\mathbf{Q}$, for which the inverse of $\mathbf{U}$, $\mathbf{U}^{-1}$, exists. The rows of $\mathbf{U}^{-1}$ are left eigenvectors of $\mathbf{Q}$.

Using the identities $\mathbf{Q} = \mathbf{UDU}^{-1}$ and $\mathbf{UU}^{-1} = \mathbf{I}$, we can rewrite the equation of state as follows

$$\frac{d}{dt}\mathbf{p} = \mathbf{pUDU}^{-1} \quad \Rightarrow \quad \frac{d}{dt}\mathbf{pU} = \mathbf{pUD}$$

Let us change co-ordinates to $\mathbf{q}(t) = \mathbf{p}(t)\mathbf{U}$. (Hence $\mathbf{p}(t) = \mathbf{q}(t)\mathbf{U}^{-1}$.) In this co-ordinate scheme,

$$\frac{d}{dt}\mathbf{q} = \mathbf{q}\mathbf{D}$$

This separates out into N independent scalar ODEs of the form

$$\frac{d}{dt}q_k(t) = \mu_k q_k(t)$$

where we recall that μ_k is the k'th eigenvalue. Clearly the solution is $q_k(t) = q_k(0)\exp(\mu_k t)$. We can write this in matrix form as $\mathbf{q}(t) = \mathbf{q}(0)\exp(\mathbf{D}t)$ if we recognise $\exp(\mathbf{D}t)$ as the diagonal matrix whose on-diagonal elements are obtained by exponentiating the diagonal elements of $\mathbf{D}t$.

We conclude this analysis by right-multiplying $\mathbf{q}(t)$ by $\mathbf{U}^{-1}$ to recover $\mathbf{p}(t) = \mathbf{q}(t)\mathbf{U}^{-1} = \mathbf{q}(0)\exp(\mathbf{D}t)\mathbf{U}^{-1} = \mathbf{p}(0)\mathbf{U}\exp(\mathbf{D}t)\mathbf{U}^{-1}$. Thus, in general,

$$\mathbf{M}(t) = \exp(\mathbf{Q}t) = \mathbf{U}\exp(\mathbf{D}t)\mathbf{U}^{-1}$$

or, in other words,

$$M_{ij}(t) = \sum_{m=1}^{N} U_{im}\exp(\mu_m t)U^{-1}_{mj} \tag{2}$$

which is the analytic result we sought.

We have chosen to obtain $\mathbf{M}(t)$ by diagonalization (as described in this section) rather than by the Padé approximant with scaling and squaring (section 3.5). We need to exponentiate a given matrix at several different timepoints t; the algebraic expression provided by diagonalization can be manipulated for increased efficiency, such as the eigenbasis projection described below. The Taylor and Padé approaches are more universal, since they do not require that the rate matrix be non-singular (have an inverse); singular rate matrices are not biologically meaningful.

3.6.1 Reversible models, equilibrium transformation and symmetry

Things become considerably simpler if $\mathbf{Q}$ is a symmetric matrix, i.e. $\mathbf{Q}^T = \mathbf{Q}$; then $\mathbf{U} = \mathbf{U^{-1}}^T$, so the left and right eigenvectors are the same. Furthermore, the eigenvalues are real (sketch of proof: let x^* be the complex conjugate of x; then show that $\sum_{ij} u_i^{(k)} Q_{ij} u_j^{(k)} = \mu_k \sum_i u_i^{(k)*} u_i^{(k)}$ is real, by taking the complex conjugate of the LHS). The eigenvectors can also be chosen to be real.

Symmetry of $\mathbf{Q}$ means that $Q_{ij} = Q_{ji}$ for all i, j. In practise, this is rare. A slightly less restrictive constraint is that $\pi_i Q_{ij} = \pi_j Q_{ji}$, where π is the *equilibrium state vector* (satisfying $\pi\mathbf{Q} = \mathbf{0}$); this is called the *detailed balance* condition and implies that the model is *time-reversible*. In this case, $\mathbf{Q}$ is related to a symmetric matrix by a simple co-ordinate transformation, so that the abovementioned benefits of a symmetric matrix can still be obtained.

Phylogeny also becomes easier for time-reversible models, since (as Felsenstein showed) we can then use the so-called *pulley principle* to slide the root node around at will, without affecting the likelihood of the tree. However, the irreversible model is more general and more realistic, and algorithms that work with an irreversible model can still be used with a reversible model.

The equilibrium co-ordinate transformation goes as follows. Let $S_{ij} = Q_{ij}\sqrt{\pi_i/\pi_j}$. We can also write this as $\mathbf{S} = \mathbf{P}^{\frac{1}{2}}\mathbf{Q}\mathbf{P}^{-\frac{1}{2}}$ where $\mathbf{P}$ is a diagonal matrix whose nonzero elements are $P_{ii} = \pi_i$. Suppose that $\mathbf{S} = \mathbf{W}\mathbf{D}\mathbf{W}^{-1}$ is the diagonalization of $\mathbf{S}$ (it's straightforward to prove that $\mathbf{S}$'s diagonal matrix $\mathbf{D}$ is the same as $\mathbf{Q}$'s). Then

$$\mathbf{W}\mathbf{D}\mathbf{W}^{-1} = \mathbf{P}^{\frac{1}{2}}\mathbf{U}\mathbf{D}\mathbf{U}^{-1}\mathbf{P}^{-\frac{1}{2}}$$

from which, by inspection, we can infer that $\mathbf{U} = \mathbf{P}^{-\frac{1}{2}}\mathbf{W}$ and $\mathbf{U}^{-1} = \mathbf{P}^{\frac{1}{2}}\mathbf{W}^{-1}$.

3.7 Trees

We now extend the treatment to phylogenetic trees, describing the pruning and peeling algorithms of Felsenstein and others. In the context of machine learning theory, these can be viewed as message-passing algorithms on a Bayesian edge-labeled graphical model (the tree itself).

The tree has K nodes, numbered $1 \ldots K$ with children lower than parents (what Knuth calls *postorder*). Thus node K is the root. Each node n of the tree has an associated state ϕ_n.

For a given node n, let $\mathcal{C}_n$ be the set of its children (if the tree is binary, this set will have zero or two members) and let (p, g) be the parent and grandparent of n, respectively. For two adjoining nodes m, n, let t_{mn} be the branch length from m to n (i.e. the evolutionary timespan separating m and n).

Let $\mathcal{T}_n$ represent the subtree containing node n and all its descendants. Let $\overline{\mathcal{T}_n}$ be the complement of $\mathcal{T}_n$, i.e. all nodes except n and its descendants.

We also have to specify some initial probability distribution over states for the root node of the tree. Let π be this *initial state vector*. For time-reversible models, we often take $\pi = \mathbf{s}$ where $\mathbf{s}$ is the equilibrium state vector, but this is not a requirement in general.

3.8 Pruning

A typical situation is that we observe the state of the leaf nodes (having performed some sequencing experiments) but we don't know the state of the ancestral internal nodes. Felsenstein's *pruning* algorithm allows us to find the likelihood of the observed data at the leaves of the tree, summing over all possible states of the missing data at the internal nodes. This is achieved by a kind of dynamic programming on the tree (it can be viewed as an instance of belief propagation on the underlying graphical model).

Define $\alpha_b^{(n)}$ to be the likelihood of the subtree $\mathcal{T}_n$ (i.e. node n and all its descendants) conditioned on node n being in state b. This likelihood is summed over all possible states at the internal nodes. Felsenstein computes this recursively

$$\alpha_b^{(n)} = \begin{cases} \delta_{bj} & \text{if } n \text{ is a leaf node in state } j \\ \displaystyle\prod_{c \in \mathcal{C}_n} \sum_k M_{bk}(t_{nc}) \alpha_k^{(c)} & \text{otherwise} \end{cases} \tag{3}$$

The $\alpha_b^{(n)}$ are computed in ascending node order, i.e. starting with $\alpha_b^{(1)}$ and ending with $\alpha_b^{(K)}$.

The summed-over-internal-states likelihood of the observed states at the leaf nodes is given by

$$\mathcal{L} = \sum_b \pi_b \alpha_b^{(K)} \tag{4}$$

3.9 Peeling

We can also find the posterior probability that a particular node (or branch) was in a particular state. Again, this is achieved by a dynamic programming algorithm, called *peeling*.

Recall that $\overline{\mathcal{T}_n}$ represents all nodes except n and its descendants. For $n < K$, $\overline{\mathcal{T}_n}$ contains at least one member: p, the parent of n.

Define $\beta_a^{(n)}$ to be the likelihood of $\overline{\mathcal{T}_n}$, again summed over all states at the internal nodes, with node p in state a.

$$\beta_a^{(n)} = \left(\prod_{\substack{c \in \mathcal{C}_p, \\ c \neq n}} \sum_j M_{aj}(t_{pc}) \alpha_j^{(c)} \right) \times \begin{cases} \pi_a & \text{if } p \text{ is root} \\ \displaystyle\sum_i \beta_i^{(p)} M_{ia}(t_{gp}) & \text{otherwise} \end{cases} \tag{5}$$

This time, the $\beta_a^{(n)}$ are filled in descending order, i.e. from root-to-leaves. Because of the fill order, we sometimes refer to the $\alpha^{(n)}$ as the "up likelihoods" and the $\beta^{(n)}$ as the "down likelihoods".

We can now write down the posterior probability $q_{ab}^{(n)}$ of a given branch (p, n) being in states (a, b)

$$q_{ab}^{(n)} = \frac{1}{\mathcal{L}} \beta_a^{(n)} M_{ab}(t_{pn}) \alpha_b^{(n)}$$

3.10 Parameter estimation and EM

Having observed a sequence alignment, and assuming some prior over trees and rate matrices, Bayes' theorem implies a posterior probability distribution over rate matrices $\mathbf{Q}$. Such a distribution can be described algebraically for pairwise sequence analysis (it's a gamma-like distribution), but gets more complex for multiple sequences. Here, we consider the simpler compromise of a single point estimate for $\mathbf{Q}$. A more rigorous inference, yielding confidence limits for $\mathbf{Q}$, can be obtained by MCMC.

The challenge we address is as follows. Given some set of observations of the state of the system at the leaves of the tree (e.g. a multiple alignment of present-day sequences), what is the best rate matrix $\mathbf{Q}$ that explains these observations?

The traditional approach (used by Dayhoff *et al* to construct the PAM matrices) is to take a pairwise alignment of two closely related sequences, count the number of instances C_{ij} of each aligned residue-pair (i, j), estimate the evolutionary distance Δt separating the two sequences, and then set $Q_{ij} \leftarrow C_{ij}/\Delta t$.

Because this approach ignores multiple substitutions at the same site (e.g. back-substitutions), it requires that only closely related sequences can be considered. (Effectively, the approach is using the discrete-time approximation introduced in section 3.4.) This means that a lot of information in the multiple alignment gets thrown away, as the sequences are too distant to consider. There are also problems with over-counting sequences.

A better, more recently popularised approach is to use *maximum likelihood* (ML); that is, to seek the rate matrix $\mathbf{Q}$ that maximises the Felsenstein likelihood of equation (4). This is the approach that will now be described.

In general, we can't write down an analytic formula for the ML rate matrix, because the choice of rate matrix $\mathbf{Q}$ affects our imputation of the state of the system at the ancestral nodes in the tree. In other words, suppose we start with some initial guess at $\mathbf{Q}$. From this, we can use the above-described peeling algorithm to make some probabilistic estimate of the missing ancestral sequences; let's call this estimate ψ. Now, given ψ, we could make an improved guess at the rate matrix; call this improved guess $\mathbf{Q}'$. However, if we then plug this new $\mathbf{Q}'$ into the peeling algorithm, we'll end up with a different estimate ψ' of the ancestral sequences. We could use this new ψ' to estimate another rate matrix $\mathbf{Q}''$, and thence another ψ'', and so on. It seems the best we can hope for is to keep iterating this procedure and hope that it converges.

It turns out that the iterative procedure just described **does** converge, and that it's a case of the *Expectation Maximisation* (EM) algorithm. In the following sections, we will describe how this works in more detail.

One should note that there are other methods of estimating rate matrices, based e.g. on *resolvents* (c.f. Vingron *et al*). We prefer EM for several reasons. First, the resolvent method is not a maximum likelihood method; it's an approximation. Second, the EM method plays better with the EM algorithms we already use to train HMMs and stochastic context-free grammars (SCFGs). Third, we think EM is more intuitive: it is basically just the same as the Dayhoff method, except that it uses unbiased posterior estimates of the counts, C_{ij} (more on this below). Fourth, EM extends naturally to ML parameterisation of more complex evolutionary models, including (for example) indel events on whole sequences.

3.11 EM approach: discrete-time case

We can get some insight into how EM works for rate matrices by revisiting the discrete-time setup of section 3.4. Recall that, in the discrete-time case, the evolutionary problem is similar to the HMM problem in that it assumes a partial observation of a discrete-time Markov process. Therefore, we can re-use the well-known version of EM for HMMs: namely, the *Baum-Welch training algorithm*.

Let's forget about phylogenetic trees for the moment, and suppose that we make two observations of the process, initially at time 0 and then at the later time $T = m\Delta t$. Let the initial observation be $\sigma(0) = a$ and the later observation be $\sigma(m) = b$.

In Baum-Welch training, the underlying iterative procedure is as follows:

- **E-step:** Given the observed data (a, b, m) and the current estimate of the (discrete-time) transition probability matrix, $\mathbf{R}$, estimate the *counts matrix* $\mathbf{C}$, where C_{ij} is the expected number of times that the Markov model made a transition from state i to state j.
- **M-step:** Set $R_{ij} \leftarrow C_{ij} / \sum_k C_{ik}$.
- Iterate until convergence.

Recall that the relationship between the discrete-time transition probability matrix $\mathbf{R}$ and the continuous-time rate matrix $\mathbf{Q}$ is (for small Δt)

$$\mathbf{R} = \mathbf{I} + \mathbf{Q}\Delta t$$

Thus, given a Baum-Welch estimate of $\mathbf{R}$, we can obtain $\mathbf{Q}$ by setting $Q_{ij} \leftarrow R_{ij}/\Delta t$ (for $i \neq j$) and $Q_{ii} = -\sum_{j \neq i} Q_{ij}$. In terms of $\mathbf{Q}$, the Baum-Welch-like algorithm for estimating transition rates therefore goes something like this:

- **E-step:** Given the observed data (a, b, m) and the current $\mathbf{Q}$, estimate the *counts matrix* $\mathbf{C}$.
- **M-step:** Set
$$Q_{ij} \leftarrow \frac{C_{ij}}{\Delta t \sum_k C_{ik}} \tag{6}$$
if $i \neq j$, and $Q_{ii} = -\sum_{j \neq i} Q_{ij}$.
- Iterate until convergence.

3.12 EM approach: continuous-time limit

Now think about what happens when we take the continuous-time limit $\Delta t \to 0$ while keeping the overall time interval T constant. The number of time-steps is $m = T/\Delta t$. Thus, as $\Delta t \to 0$, we have $m \to \infty$: as the size of the time-step tends to zero, the number of discrete steps required to span the time interval tends to infinity.

For two distinct states i, j (with $i \neq j$), the expected number C_{ij} of $i \to j$ transitions should not depend too much on Δt. This is because C_{ij} represents something "real" (the number of times that an i mutated to a j), which is independent of the time-step.

However, if we look at the expected number of self-transitions, C_{ii}, we find a problem. Namely, $C_{ii} \sim T/\Delta t$, so that as $\Delta t \to 0$, the number of self-transitions will blow up. This is because C_{ii} does not represent a "real" quantity. What it represents is the number of time-steps during which the system stayed in state i and did not mutate, and this totally depends on the size we have chosen for the time-step, which of course is an artificial quantity (and one we hope to eliminate).

To fix this, let us define $w_i = \Delta t C_{ii}$, the expected amount of time that the system spent in state i. Let's call this the *wait time* for state i. We expect that the wait time will not, ultimately, depend on Δt.

If we now look at the denominator on the right-hand-side of equation (6), we see that the continuous-time limit $\lim_{\Delta t \to 0} \Delta t C_{ik}$ is equal to 0 if $i \neq k$, and w_i if $i = k$. In other words, the continuous-time limit of the Baum-Welch M-step is

$$Q_{ij} \leftarrow \frac{C_{ij}}{w_i}$$

if $i \neq j$, with $Q_{ii} = -\sum_{j \neq i} C_{ij}/w_i$.

The interpretation of this is that the best estimate for the rate from i to j is equal to C_{ij}, the expected number of $i \to j$ mutations, divided by w_i, the expected amount of time spent in state i. This is exactly equivalent to the Dayhoff approach, except that C_{ij} and w_i are now unbiased posterior estimates of the mutation count and the wait time, rather than naive (biased) counts.

3.13 Estimating the counts and the wait times

It remains to be shown how to estimate the mutation counts $\mathbf{C}$ and wait times $\mathbf{w}$. In this section we show how to do this for a single pair of observations using the eigenvector decomposition of $\mathbf{Q}$.

Recall our observed data are $\sigma(0) = a$ and $\sigma(T) = b$. The likelihood of such an observation is $M_{ab}(T)$.

Suppose that a mutation $i \to j$ occurs at a particular time t, where $0 \leq t \leq T$. The probability of going from a to i in time t is $M_{ai}(t)$; the probability of the mutation $i \to j$ occuring in a time interval of size dt is $Q_{ij}dt$; and the probability of going from j to b in time $T - t$ is $M_{jb}(t)$. Dividing by $M_{ab}(T)$ to obtain the posterior probability of this mutation, and integrating over all times t, we obtain

$$C_{ij} = \frac{1}{M_{ab}(T)} \int_0^T M_{ai}(t) Q_{ij} M_{jb}(T-t) dt$$

Now suppose that the process is in state i at time t, and no mutation occurs. The probability of going from a to i in time t is $M_{ai}(t)$ and the probability of going from i to b in time $T - t$ is $M_{ib}(t)$. This instant contributes dt to the total wait-time in state i. Therefore

$$w_i = \frac{1}{M_{ab}(T)} \int_0^T M_{ai}(t) M_{ib}(T-t) dt$$

We can write these results as

$$C_{ij} = \frac{Q_{ij}}{M_{ab}(T)} \mathcal{I}_{ij}^{ab}(T) \ , \qquad\qquad w_i = \frac{C_{ii}}{Q_{ii}}$$

where we have introduced the new function $\mathcal{I}_{ij}^{ab}(T) = \int_0^T M_{ai}(t) M_{jb}(T-t) dt$. Plugging in the definition of $M_{ij}(t)$ from equation (2), we obtain

$$\mathcal{I}_{ij}^{ab}(T) = \sum_{k=1}^{N} U_{ak} U_{ki}^{-1} \sum_{l=1}^{N} U_{jl} U_{lb}^{-1} \mathcal{J}_{kl}(T) \qquad (7)$$

where $\mathcal{J}(T)$ is the following kernel matrix

$$\mathcal{J}_{kl}(T) \quad = \quad \begin{array}{cl} T \exp(\mu_k T) & \text{if } \mu_k = \mu_l \\ \frac{\exp(\mu_k T) - \exp(\mu_l T)}{\mu_k - \mu_l} & \text{if } \mu_k \neq \mu_l \end{array} \qquad (8)$$

3.14 Putting it together: EM for rates on trees

In this section, we outline how to combine the results of sections 3.9, 3.12 and 3.13 to estimate $\mathbf{Q}$ by EM from a multiple alignment.

The algorithm proceeds as follows.

- Set $\mathbf{C} \leftarrow \mathbf{0}$.
- For each column c in the multiple alignment:
  - Use the peeling algorithm of section 3.8 to compute the posterior probability $q_{ab}^{(n)}$ for each adjoining pair of nodes (p, n) of the tree being in states (a, b).
  - For each branch (p, n) with length $T = T_{pn}$ and each $a, b, i, j \in \{1 \ldots N\}$:
    - Set $C_{ij} \leftarrow C_{ij} + q_{ab}^{(n)} \frac{Q_{ij}}{M_{ab}(T)} \mathcal{I}_{ij}^{ab}(T)$.
- Calculate the next estimate of the rate matrix, $\mathbf{Q}'$, as follows:
  - Let $w_i = C_{ii}/Q_{ii}$.
  - For all $i, j \neq i$, set $Q'_{ij} \leftarrow C_{ij}/w_i$.

– For all i, set $Q'_{ii} \leftarrow -(\sum_{j \neq i} C_{ij})/w_i$.

- Set $\mathbf{Q} \leftarrow \mathbf{Q}'$.
- Repeat until the joint likelihood, $\mathcal{L}$, converges.

As with the Baum-Welch algorithm, we can inform the parameter estimation using prior distributions. With Baum-Welch, one typically uses (mixtures of) Dirichlet distributions, as these are naturally conjugate to the multinomial distribution underlying the counts. With the rate EM algorithm, the natural conjugate priors are (mixtures of) gamma distributions over the Q_{ij} (which can, equivalently, be viewed as a gamma distribution for the exit rates $-Q_{ii}$ combined with a Dirichlet distribution over the probabilities $-Q_{ij}/Q_{ii}$).

3.15 Accumulating the counts in eigenvector space

The inner loop of the algorithm in section 3.14 is over four state variables (a, b, i, j) each of which takes N values. If $\mathcal{I}_{ij}^{ab}(T_{pn})$ is not cached (which would take $O(N^4)$ memory per branch and so is impractical) then a further loop over two state variables (m, n) is required. The complexity of all of these loops together is $O(N^6)$, as is evident from the following expanded expression for C_{ij}

$$C_{ij} = \sum_{\mathcal{A}} \sum_{c \in \mathcal{A}} \sum_{(p,n) \in \mathcal{T}(c)} \sum_{a,b}^{N} q_{ab}^{(n)} \frac{Q_{ij}}{M_{ab}(T_{pn})} \sum_{k}^{N} U_{ak} U_{ki}^{-1} \sum_{l}^{N} U_{jl} U_{lb}^{-1} \mathcal{J}_{kl}(T_{pn})$$

where $\mathcal{A}$ represents each multiple alignment, c each column of $\mathcal{A}$ and $\mathcal{T}(c)$ the tree of states corresponding to column c.

We can't eliminate this $O(N^6)$ time hit, but we can rearrange the sums in a more efficient form.

$$C_{ij} = \sum_{k}^{N} U_{ki}^{-1} \sum_{l}^{N} U_{jl} \left(\sum_{\mathcal{A}} \sum_{c \in \mathcal{A}} \sum_{(p,n) \in \mathcal{T}(c)} \sum_{a,b}^{N} q_{ab}^{(n)} \frac{Q_{ij}}{M_{ab}(T_{pn})} U_{ak} U_{lb}^{-1} \mathcal{J}_{kl}(T_{pn}) \right)$$

This can be described as accumulating the counts in eigenvector space, then transforming back. Finally we can use the expression for $q_{ab}^{(n)}$

$$q_{ab}^{(n)} = \frac{1}{\mathcal{L}_c} \beta_a^{(n)} M_{ab}(T_{pn}) \alpha_b^{(n)}$$

where $\mathcal{L}_c$ is the likelihood of column c. Substituting this into the equation for C_{ij}, and rearranging, gives

$$C_{ij} = Q_{ij} \sum_{k}^{N} U_{ki}^{-1} \sum_{l}^{N} U_{jl} \left(\sum_{\mathcal{A}} \sum_{c \in \mathcal{A}} \frac{1}{\mathcal{L}_c} \sum_{(p,n) \in \mathcal{T}(c)} \mathcal{D}_k^{(n)} \ \mathcal{J}_{kl}(T_{pn}) \ \mathcal{U}_l^{(n)} \right)$$

where $\mathcal{U}$ and $\mathcal{D}$ are (respectively) the left and right eigenbasis projections of the "up" and "down" peeling likelihoods, α and β.

$$\mathcal{U}_l^{(n)} = \sum_{b=1}^{N} \alpha_b^{(n)} U_{lb}^{-1} \quad \mathcal{D}_k^{(n)} = \sum_{a=1}^{N} \beta_a^{(n)} U_{ak}$$

In practice the projections $\mathcal{U}$ and $\mathcal{D}$ are calculated at the same time that the peeling likelihoods α and β are; the tree need only be traversed up and down once per EM cycle.

Finally, we can rewrite the expression for C_{ij} as

$$C_{ij} = Q_{ij} \sum_{k}^{N} U_{ki}^{-1} \sum_{l}^{N} U_{jl} E_{kl} \quad (9)$$

$$E_{kl} = \sum_{\mathcal{A}} \sum_{c \in \mathcal{A}} \frac{1}{\mathcal{L}_c} \sum_{(p,n) \in \mathcal{T}(c)} \mathcal{D}_k^{(n)} \ \mathcal{J}_{kl}(T_{pn}) \ \mathcal{U}_l^{(n)} \quad (10)$$

where $\mathbf{E}$ is the matrix of "eigencounts".

3.16 Complex eigensystems

Recall that the characteristic equation

$$|\mathbf{Q} - \mu \mathbf{I}| = 0$$

is a degree-N polynomial with real coefficients (since the Q_{ij}) are real.

It follows that the roots of this polynomial must either be real, or complex conjugate pairs (sketch of proof: complex conjugativity is distributive over multiplication and addition, therefore if x is a zero of the characteristic then x^* must also be a zero. Intuitively, there is no way the real coefficients can "distinguish" between two conjugate roots $\pm\sqrt{-1}$.)

Let k be the index of an eigenvalue, μ_k. If μ_k is complex, then let the index of the conjugate eigenvalue be k'. Likewise let l be the index of a (possibly different) eigenvalue and l' the index of the conjugate (if μ_l is complex). Since each of μ_k and μ_l can be either real or complex, there are four possibilities for complex conjugate symmetries:

Condition	Eigenvalues	Eigenvectors	Eigenprojections	Kernel	Eigencounts	Summary
μ_k real,<br>μ_l real;	(both real)	U_{ik}, U^{-1}_{ki} real<br>U_{il}, U^{-1}_{li} real	$\mathcal{U}^{(n)}_l$ real<br>$\mathcal{D}^{(n)}_k$ real	$\mathcal{J}_{kl}$ real	E_{kl} real	(real)
μ_k real,<br>μ_l complex;	$\mu_l = \mu^*_{l'}$	U_{ik}, U^{-1}_{ki} real<br>$U_{il} = U^*_{il'}$<br>$U^{-1}_{li} = (U^{-1}_{l'i})^*$	$\mathcal{U}^{(n)}_l = (\mathcal{U}^{(n)}_{l'})^*$<br>$\mathcal{D}^{(n)}_k$ real	$\mathcal{J}_{kl} = (\mathcal{J}_{kl'})^*$	$E_{kl} = (E_{kl'})^*$	$l \leftrightarrow l'$<br>$(k,l) \leftrightarrow (k,l')$
μ_k complex,<br>μ_l real;	$\mu_k = \mu^*_{k'}$	$U_{ik} = U^*_{ik'}$<br>$U^{-1}_{ki} = (U^{-1}_{k'i})^*$<br>$U_{il}, U^{-1}_{li}$ real	$\mathcal{U}^{(n)}_l$ real<br>$\mathcal{D}^{(n)}_k = (\mathcal{D}^{(n)}_{k'})^*$	$\mathcal{J}_{kl} = (\mathcal{J}_{k'l})^*$	$E_{kl} = (E_{k'l})^*$	$k \leftrightarrow k'$<br>$(k,l) \leftrightarrow (k',l)$
μ_k complex,<br>μ_l complex.	$\mu_k = \mu^*_{k'}$<br>$\mu_l = \mu^*_{l'}$	$U_{ik} = U^*_{ik'}$<br>$U^{-1}_{ki} = (U^{-1}_{k'i})^*$<br>$U_{il} = U^*_{il'}$<br>$U^{-1}_{li} = (U^{-1}_{l'i})^*$	$\mathcal{U}^{(n)}_l = (\mathcal{U}^{(n)}_{l'})^*$<br>$\mathcal{D}^{(n)}_k = (\mathcal{D}^{(n)}_{k'})^*$	$\mathcal{J}_{kl} = (\mathcal{J}_{k'l'})^*$<br>$\mathcal{J}_{k'l} = (\mathcal{J}_{kl'})^*$	$E_{kl} = (E_{k'l'})^*$<br>$E_{k'l} = (E_{kl'})^*$	$k \leftrightarrow k'$<br>$l \leftrightarrow l'$<br>$(k,l) \leftrightarrow (k',l')$<br>$(k',l) \leftrightarrow (k,l')$

Write the following complex expansions

$$\begin{aligned} \mu_k &= \kappa_k + \imath \lambda_k \\ \mathbf{U} &= \mathbf{V} + \imath \mathbf{W} \\ \mathbf{U}^{-1} &= \mathbf{X} + \imath \mathbf{Y} \end{aligned}$$

where $\imath = \sqrt{-1}$, κ_k, λ_k are real and $\mathbf{V}, \mathbf{W}, \mathbf{X}$ and $\mathbf{Y}$ are real $N \times N$ matrices.

Using the identities $(xyz)^* = x^* y^* z^*$ and $x + x^* = 2\mathrm{Re}\,[x]$, we can write equation (2) as

$$\begin{aligned} M_{ij}(t) &= \sum_{m=1}^{N} U_{im} \exp(\mu_n t) U^{-1}_{mj} \\ &= \sum_{m:\lambda_m=0} V_{im} \exp(\kappa_m t) X_{mj} + 2\mathrm{Re}\left[\sum_{m:\lambda_m>0} (V_{im} + \imath W_{im}) \exp((\kappa_m + \imath\lambda_m)t)(X_{mj} + \imath Y_{mj}) \right] \\ &= \sum_{m:\lambda_m=0} V_{im} c_m X_{mj} + 2 \sum_{m:\lambda_m>0} \left(c_m (V_{im} X_{mj} - W_{im} Y_{mj}) - s_m (V_{im} Y_{mj} + W_{im} X_{mj}) \right) \qquad (11) \end{aligned}$$

where

$$c_k = \exp(\kappa_k T)\cos(\lambda_k T) \text{ and } s_k = \exp(\kappa_k T)\sin(\lambda_k T) \tag{12}$$

The complex form of $\mathcal{J}_{kl}(T)$, from equation (8), is as follows. For repeated eigenvalues, $\mu_k = \mu_l$:

$$\begin{aligned}\mathcal{J}_{kl}(T) &= T\exp(\mu_k T)\\ &= T(c_k + \imath s_k)\end{aligned}$$

For unique eigenvalues, $\mu_k \neq \mu_l$:

$$\begin{aligned}\mathcal{J}_{kl}(T) &= \frac{\exp(\mu_k T) - \exp(\mu_l T)}{\mu_k - \mu_l}\\ &= \frac{(c_k + \imath s_k) - (c_l + \imath s_l)}{(\kappa_k + \imath\lambda_k) - (\kappa_l + \imath\lambda_l)}\\ &= \frac{((c_k - c_l) + \imath(s_k - s_l))((\kappa_k - \kappa_l) - \imath(\lambda_k - \lambda_l))}{(\kappa_k - \kappa_l)^2 + (\lambda_k - \lambda_l)^2}\\ &= \frac{((c_k - c_l)(\kappa_k - \kappa_l) + (s_k - s_l)(\lambda_k - \lambda_l)) + \imath((s_k - s_l)(\kappa_k - \kappa_l) - (c_k - c_l)(\lambda_k - \lambda_l))}{(\kappa_k - \kappa_l)^2 + (\lambda_k - \lambda_l)^2}\\ &= \frac{(c_{kl}\kappa_{kl} + s_{kl}\lambda_{kl}) + \imath(s_{kl}\kappa_{kl} - c_{kl}\lambda_{kl})}{\kappa_{kl}^2 + \lambda_{kl}^2}\end{aligned}$$

where $c_{kl} = c_k - c_l$, $s_{kl} = s_k - s_l$, $\kappa_{kl} = \kappa_k - \kappa_l$ and $\lambda_{kl} = \lambda_k - \lambda_l$.

3.17 Implementation of complex matrix algebra

The following is a fairly detailed discussion of an implementation of the concepts described above. This section may not be of great interest to a general audience; it was written as a guide to aid in code maintenance.

Our implementation has made heavy use of Gerton Lunter's EISPACK-based matrix diagonalization and matrix exponentiation code. This code returns the eigenvalues and eigenvectors in a condensed fashion, implicitly using the fact that complex eigenvalues of real matrices come in conjugate pairs. The eigenvalues are contained in a single vector; for complex eigenvalue μ_k, the real part of the pair is in position k and the positive imaginary part in position $k+1$. The corresponding pair of conjugate (right) eigenvectors are similarly returned in columns k (real part) and $k+1$ (positive imaginary part) of the eigenvector matrix $\mathbf{U}$.

The second matrix returned, $\mathbf{U}^{-1}$, which in the real case is the inverse of the eigenvector matrix and contains left eigenvectors as rows, does not directly correspond to the complex matrix inverse; it is simply the inverse of the eigenvector matrix, viewed as real. The procedure to determine the actual complex inverse matrix from $\mathbf{U}^{-1}$ is straightforward. Rows corresponding to real eigenvalues μ_k are correct. As before, rows corresponding to complex eigenvalues μ_k are treated as pairs, k and $k+1$, with row k containing the real and $k+1$ the imaginary part. In addition, these real and imaginary parts must be divided by two; the sign of the imaginary part must also be reversed, *i.e.*, the imaginary value in row $k+1$ corresponds to the eigenvalue with negative imaginary part. This translation procedure is easily validated by calculating the product matrix, $(\mathbf{U}\mathbf{U}^{-1})_{ik} = \sum_{j=1}^{N} \mathbf{U}_{ij}\mathbf{U}^{-1}_{jk}$ using the compaction rules. Entries corresponding to real eigenvalues contribute $\mathbf{U}_{ij}\mathbf{U}^{-1}_{jk}$; entries corresponding to a conjugate pair of eigenvalues j and $j+1$ contribute $2\mathbf{U}_{ij}\mathbf{U}^{-1}_{jk} - 2\mathbf{U}_{i,j+1}\mathbf{U}^{-1}_{j+1,k}$, which gives the desired result upon dividing by 2 and reversing the sign of $\mathbf{U}^{-1}_{j+1,k}$.

The following describes how we use this code in calculating the matrix exponential, equation (2), making use of an intermediate matrix Z_{mj} as defined here. This procedure is equivalent to equation(11) after

converting $\mathbf{U}^{-1}$ to the actual complex inverse.

$$\begin{aligned} M_{ij}(t) &= \textstyle\sum_{m=1}^{N} U_{im} Z_{mj} \\ Z_{mj} &= \exp(\mu_m t) U^{-1}_{mj} && \text{eigenvalue } \mu_m \text{ real} \\ Z_{mj} &= c_m U^{-1}_{mj} + s_m U^{-1}_{m+1,j} && \text{eigenvalue } \mu_m \text{ complex} \\ Z_{m+1,j} &= -s_m U^{-1}_{mj} + c_m U^{-1}_{m+1,j} && \text{''} \end{aligned}$$

with c_m and s_m as defined in equation (12).

We also need to calculate the complex form of the "eigencounts" matrix $\mathbf{E}$. The complex forms of the left and right eigenbasis projections $\mathcal{U}$ and $\mathcal{D}$ of the "up" and "down" peeling likelihoods α and β, described in section 3.15, and the kernel matrix $\mathcal{J}_{kl}$, are written as follows:

$$\mathcal{U}^{(n)}_l = \mathcal{U}^{r(n)}_l + \imath \mathcal{U}^{i(n)}_l \quad \mathcal{D}^{(n)}_k = \mathcal{D}^{r(n)}_k + \imath \mathcal{D}^{i(n)}_k \quad \mathcal{J}_{kl} = \mathcal{J}^r_{kl} + \imath \mathcal{J}^i_{kl}$$

where $\mathcal{U}^{r(n)}_l$, $\mathcal{U}^{i(n)}_l$, $\mathcal{D}^{r(n)}_k$, $\mathcal{D}^{i(n)}_k$, $\mathcal{J}^r_{kl}$ and $\mathcal{J}^i_{kl}$ are all real.

The complex form of equation (10) is

$$\begin{aligned} E_{kl} = &= \sum_{\mathcal{A}} \sum_{c \in \mathcal{A}} \frac{1}{\mathcal{L}_c} \sum_{(p,n) \in \mathcal{T}(c)} (\mathcal{D}^{r(n)}_k + \imath \mathcal{D}^{i(n)}_k) \, (\mathcal{J}^r_{kl}(T_{pn}) + \imath \mathcal{J}^i_{kl}(T_{pn})) \, (\mathcal{U}^{r(n)}_l + \imath \mathcal{U}^{i(n)}_l) \\ &= \sum_{\mathcal{A}} \sum_{c \in \mathcal{A}} \frac{1}{\mathcal{L}_c} \sum_{(p,n) \in \mathcal{T}(c)} \left(\begin{array}{c} \mathcal{D}^{r(n)}_k [\mathcal{J}^r_{kl}(T_{pn}) \mathcal{U}^{r(n)}_l - \mathcal{J}^i_{kl}(T_{pn}) \mathcal{U}^{i(n)}_l] - \mathcal{D}^{i(n)}_k [\mathcal{J}^r_{kl} \mathcal{U}^{i(n)}_l + \mathcal{J}^i_{kl} \mathcal{U}^{r(n)}_l] \\ + \imath \left\{ \mathcal{D}^{i(n)}_k [\mathcal{J}^r_{kl}(T_{pn}) \mathcal{U}^{r(n)}_l - \mathcal{J}^i_{kl}(T_{pn}) \mathcal{U}^{i(n)}_l] + \mathcal{D}^{r(n)}_k [\mathcal{J}^r_{kl} \mathcal{U}^{i(n)}_l + \mathcal{J}^i_{kl} \mathcal{U}^{r(n)}_l] \right\} \end{array} \right) \end{aligned}$$

The conjugate symmetries make it both possible and advantageous to store the complex vectors and arrays in condensed form; in the vectors the entries corresponding to complex eigenvalue μ_k have real part in position k and the imaginary part corresponding to the eigenvalue with positive imaginary part in position $k+1$. The complex arrays $\mathcal{J}_{kl}(T)$ and E_{kl} have slightly more interesting conjugate symmetry in the case where both μ_k and μ_l are complex. Here, *e.g.* for E_{kl}, the conjugate pairs are $E_{kl} \leftrightarrow E_{k+1,l+1}$ and $E_{k+1,l} \leftrightarrow E_{k,l+1}$. We adopt the convention that, in the (real) tables used in the implementation, entries E_{kl} and $E_{k+1,l}$ contain the real part of the pair; entries $E_{k+1,l+1}$ and $E_{k,l+1}$ contain the imaginary part corresponding to the eigenvalue with positive imaginary part.

The implementation includes four distinct cases, one for each of the possible combinations of real and complex μ_k and μ_l. If both are real, there is only one corresponding E_{kl} table entry:

$$E_{kl} = \sum_{\mathcal{A}} \sum_{c \in \mathcal{A}} \frac{1}{\mathcal{L}_c} \sum_{(p,n) \in \mathcal{T}(c)} \mathcal{D}^{(n)}_k \mathcal{J}_{kl}(T_{pn}) \mathcal{U}^{(n)}_l$$

If μ_k is real and μ_l is complex, we calculate two table entries together: E_{kl}, containing the real part of the corresponding conjugate pair, and $E_{k,l+1}$, containing the imaginary part of the complex entry corresponding to the eigenvalue μ_l with positive imaginary part:

$$\begin{aligned} E_{kl} &= \sum_{\mathcal{A}} \sum_{c \in \mathcal{A}} \frac{1}{\mathcal{L}_c} \sum_{(p,n) \in \mathcal{T}(c)} \mathcal{D}^{(n)}_k (\mathcal{J}_{kl}(T_{pn}) \mathcal{U}^{(n)}_l - \mathcal{J}_{k,l+1}(T_{pn}) \mathcal{U}^{(n)}_{l+1}) \\ E_{k,l+1} &= \sum_{\mathcal{A}} \sum_{c \in \mathcal{A}} \frac{1}{\mathcal{L}_c} \sum_{(p,n) \in \mathcal{T}(c)} \mathcal{D}^{(n)}_k (\mathcal{J}_{kl}(T_{pn}) \mathcal{U}^{(n)}_{l+1} + \mathcal{J}_{k,l+1}(T_{pn}) \mathcal{U}^{(n)}_l) \end{aligned}$$

As one might expect, the case where μ_k is complex and μ_l is real is similar:

$$E_{kl} = \sum_{\mathcal{A}} \sum_{c \in \mathcal{A}} \frac{1}{\mathcal{L}_c} \sum_{(p,n) \in \mathcal{T}(c)} (\mathcal{D}_k^{(n)} \mathcal{J}_{kl}(T_{pn}) - \mathcal{D}_{k+1}^{(n)} \mathcal{J}_{k+1,l}(T_{pn})) \mathcal{U}_l^{(n)}$$

$$E_{k+1,l} = \sum_{\mathcal{A}} \sum_{c \in \mathcal{A}} \frac{1}{\mathcal{L}_c} \sum_{(p,n) \in \mathcal{T}(c)} (\mathcal{D}_{k+1}^{(n)} \mathcal{J}_{kl}(T_{pn}) + \mathcal{D}_k^{(n)} \mathcal{J}_{k+1,l}(T_{pn})) \mathcal{U}_l^{(n)}$$

In the case where μ_k and μ_l are both complex, there are two real parts: E_{kl} for the $E_{kl} \leftrightarrow E_{k+1,l+1}$ pair and $E_{k+1,l}$ for the $E_{k+1,l} \leftrightarrow E_{k,l+1}$ pair, and two imaginary parts: $E_{k+1,l+1}$ for the former pair and $E_{k,l+1}$ for the latter.

$$E_{kl} = \sum_{\mathcal{A}} \sum_{c \in \mathcal{A}} \frac{1}{\mathcal{L}_c} \sum_{(p,n) \in \mathcal{T}(c)} \mathcal{D}_k^{(n)} (\mathcal{J}_{kl}(T_{pn}) \mathcal{U}_l^{(n)} - \mathcal{J}_{k+1,l+1}(T_{pn}) \mathcal{U}_{l+1}^{(n)}) - \mathcal{D}_{k+1}^{(n)} (\mathcal{J}_{kl}(T_{pn}) \mathcal{U}_{l+1}^{(n)} + \mathcal{J}_{k+1,l+1}(T_{pn}) \mathcal{U}_l^{(n)})$$

$$E_{k+1,l} = \sum_{\mathcal{A}} \sum_{c \in \mathcal{A}} \frac{1}{\mathcal{L}_c} \sum_{(p,n) \in \mathcal{T}(c)} \mathcal{D}_k^{(n)} (\mathcal{J}_{kl}(T_{pn}) \mathcal{U}_l^{(n)} - \mathcal{J}_{k,l+1}(T_{pn}) \mathcal{U}_{l+1}^{(n)}) + \mathcal{D}_{k+1}^{(n)} (\mathcal{J}_{kl}(T_{pn}) \mathcal{U}_{l+1}^{(n)} + \mathcal{J}_{k,l+1}(T_{pn}) \mathcal{U}_l^{(n)})$$

$$E_{k,l+1} = \sum_{\mathcal{A}} \sum_{c \in \mathcal{A}} \frac{1}{\mathcal{L}_c} \sum_{(p,n) \in \mathcal{T}(c)} \mathcal{D}_{k+1}^{(n)} (\mathcal{J}_{kl}(T_{pn}) \mathcal{U}_l^{(n)} - \mathcal{J}_{k,l+1}(T_{pn}) \mathcal{U}_{l+1}^{(n)}) - \mathcal{D}_k^{(n)} (\mathcal{J}_{kl}(T_{pn}) \mathcal{U}_{l+1}^{(n)} + \mathcal{J}_{k,l+1}(T_{pn}) \mathcal{U}_l^{(n)})$$

$$E_{k+1,l+1} = \sum_{\mathcal{A}} \sum_{c \in \mathcal{A}} \frac{1}{\mathcal{L}_c} \sum_{(p,n) \in \mathcal{T}(c)} \mathcal{D}_{k+1}^{(n)} (\mathcal{J}_{kl}(T_{pn}) \mathcal{U}_l^{(n)} - \mathcal{J}_{k+1,l+1}(T_{pn}) \mathcal{U}_{l+1}^{(n)}) + \mathcal{D}_k^{(n)} (\mathcal{J}_{kl}(T_{pn}) \mathcal{U}_{l+1}^{(n)} + \mathcal{J}_{k+1,l+1}(T_{pn}) \mathcal{U}_l^{(n)})$$

In the final calculation, of C_{ij}, the conjugate symmetries lead to a simpler form. Since the sums in equation (9) include both entries of conjugate pairs, the imaginary part is zero, as expected for transition counts. In practice, the double sum (9) is calculated as a single nested sum

$$C_{ij} = Q_{ij} \sum_k^N \sum_l^N U_{ki}^{-1} E_{kl} U_{jl} \tag{13}$$

Because the elements of the inverse matrix $\mathbf{U}^{-1}$ are left eigenvectors as rows, the $\mathbf{U}_{ki}^{-1}$ entries for a given k contain either strictly real elements, in the case of a real eigenvector μ_k, or either the real part (row k) or the imaginary part (row $k+1$) in the case of a complex μ_k, regardless of subscript i. Similarly the elements of matrix $\mathbf{U}$ are (right) eigenvectors as columns, so the $\mathbf{U}_{jl}$ entries for a given l are also either strictly real, for real μ_l, or either the real or imaginary part of a complex pair l and $l+1$ for complex μ_l, regardless of subscript j. This means that the evaluation of expression (13) need only be concerned with whether indices k and l correspond to real or complex μ_k or μ_l and not with i or j.

In evaluating the sum, we again have four distinct cases. If both μ_k and μ_l are real, we include in the sum only the term:

$$U_{ki}^{-1} E_{kl} U_{jl}$$

For μ_k real and μ_l complex, we calculate terms for indices l and $l+1$ together, which contribute the following (remembering that we have taken into account the relationship between the U_{ki}^{-1} entries and the actual complex matrix inverse, which here removes a factor of two):

$$U_{ki}^{-1} (E_{kl} U_{jl} - E_{k,l+1} U_{j,l+1})$$

The expression for μ_k complex and μ_l real is similar; here we process terms k and $k+1$ together. The translation of the $U_{k+1,i}^{-1}$ entry reverses the sign of the second term:

$$(U_{ki}^{-1} E_{kl} + U_{k+1,i}^{-1} E_{k+1,l}) U_{jl}$$

In the calculation in the case where both μ_k and μ_l are complex, we process together terms k and $k+1$, l and $l+1$, which together give:

$$U_{ki}^{-1} (E_{kl}^r U_{jl} - E_{kl}^i U_{j,l+1}) + U_{k+1,i}^{-1} (E_{kl}^i U_{jl} + E_{kl}^r U_{j,l+1})$$

where E^r_{kl} and E^i_{kl} are the sum of the real and imaginary E_{kl} terms, respectively:

$$E^r_{kl} = E_{kl} + E_{k+1,l} \quad E^i_{kl} = E_{k+1,l+1} + E_{k,l+1}$$

In this calculation the pesky signs are easily validated: the product of two imaginary terms gives a factor of -1, as does the term $U^{-1}_{k+1,i}$.

3.18 Application to phylo-grammars

Notationally, the application of the above EM algorithm to phylo-grammars is slightly involved, but the gist is as follows.

The "standard" biological formulation, as described in e.g. the textbook by Durbin *et al*, of the dynamic programming algorithm for aligning sequences to a hidden Markov model (HMM) or stochastic context-free grammar (SCFG), can be sketched (non-rigorously) as follows:

- For each subsequence S of the full sequence, iterating from shortest to longest:
  - For each nonterminal ϕ in the grammar:
    - Let E denote the final residue(s) of S and let S' denote the remainder of S.
    - $(*)$ Calculate the likelihood of emitting residues E from state ϕ.
    - Multiply this by the max (or sum) of all incoming transitions from S'.
    - Store this result in cell (ϕ, S).

Note that the emission E is not restricted to be a single residue. Examples of models that typically make use of multi-residue emissions are RNA structure (the two nucleotides of a base-pair) and protein-coding genes (the three nucleotides of a codon). In the single-sequence grammar, it is not essential to aggregate these emissions to a single state: one can achieve the same effect by staggering the multi-residue emission over several states (an emission of N correlated residues from an alphabet of size A requires $\sim A^{N-1}$ "memory" states, each emitting one symbol). This trick doesn't work for phylo-grammars, where we are interested in the likelihood of not just one N-mer but rather multiple observations of this N-mer in homologous sequences, correlated according to some underlying phylogenetic tree. Thus, while many implementations of single-sequence grammars allow at most one residue to be emitted per state without loss of generality (see e.g. Durbin *et al*, "Biological Sequence Analysis"). implementations of phylo-grammars must allow multi-residue emissions.

To adapt the above kind of algorithm for phylo-grammars, it suffices merely to modify the emit likelihood calculation, flagged with an asterisk $(*)$ in the above text. Specifically, the emission E is no longer a residue (or residues), but rather an alignment column (or columns). Instead of calculating the likelihood of emitting a single symbol (or a set of covariant symbols) one must now calculate the likelihood of an entire alignment column (or set of covariant columns). This is easily achieved using the phylogenetic "pruning" algorithms of section 3.7.

The Inside-Outside and Forward-Backward algorithms are similarly easy to modify. Indeed the calculation of posterior probabilities is unchanged; it is only the accumulation of counts that must be modified. Again sacrificing exactness for brevity, the single-sequence case may be written as follows:

- Let $C(\phi, E)$ be the expected number of times that state ϕ emitted residue(s) E.
- Initialise all $C(\phi, E) \leftarrow 0$.
- For each subsequence S of the full sequence:
  - For each nonterminal ϕ in the grammar:
    - Let E denote the final residue(s) of S and let S' denote the remainder of S.
    - Calculate p, the posterior probability that E was emitted by state ϕ.
    - $(*)$ Set $C(\phi, E) \leftarrow C(\phi, E) + p$.

The step that must be modified is again flagged with an asterisk (∗). Again, for the phylo-grammar E represents a column (or set of columns) rather than a single residue (or set of residues). Rather than simply incrementing a single count, one now runs the phylo-EM algorithm of section 3.13 on column E in order to estimate a full set of expected substitution transitions and wait times (the C_{ij} and w_i of section 3.13). These counts, weighted by the posterior probability p, are then added to running totals for C_{ij} and w_i for state ϕ. These running totals are used to update the rate matrix for ϕ in the M-step of EM.
